# HER2+ Cancer Cell Dependence on PI3K *vs*. MAPK Signaling Axes Is Determined by Expression of EGFR, ERBB3 and CDKN1B

**DOI:** 10.1371/journal.pcbi.1004827

**Published:** 2016-04-01

**Authors:** Daniel C. Kirouac, Jinyan Du, Johanna Lahdenranta, Matthew D. Onsum, Ulrik B. Nielsen, Birgit Schoeberl, Charlotte F. McDonagh

**Affiliations:** Discovery, Merrimack Pharmaceuticals, Cambridge, Massachusetts, United States of America; Johns Hopkins University, UNITED STATES

## Abstract

Understanding the molecular pathways by which oncogenes drive cancerous cell growth, and how dependence on such pathways varies between tumors could be highly valuable for the design of anti-cancer treatment strategies. In this work we study how dependence upon the canonical PI3K and MAPK cascades varies across HER2+ cancers, and define biomarkers predictive of pathway dependencies. A panel of 18 HER2+ (*ERBB2*-amplified) cell lines representing a variety of indications was used to characterize the functional and molecular diversity within this oncogene-defined cancer. PI3K and MAPK-pathway dependencies were quantified by measuring *in vitro* cell growth responses to combinations of AKT (MK2206) and MEK (GSK1120212; trametinib) inhibitors, in the presence and absence of the ERBB3 ligand heregulin (NRG1). A combination of three protein measurements comprising the receptors EGFR, ERBB3 (HER3), and the cyclin-dependent kinase inhibitor p27 (CDKN1B) was found to accurately predict dependence on PI3K/AKT vs. MAPK/ERK signaling axes. Notably, this multivariate classifier outperformed the more intuitive and clinically employed metrics, such as expression of phospho-AKT and phospho-ERK, and PI3K pathway mutations (*PIK3CA*, *PTEN*, and *PIK3R1*). In both cell lines and primary patient samples, we observed consistent expression patterns of these biomarkers varies by cancer indication, such that ERBB3 and CDKN1B expression are relatively high in breast tumors while EGFR expression is relatively high in other indications. The predictability of the three protein biomarkers for differentiating PI3K/AKT vs. MAPK dependence in HER2+ cancers was confirmed using external datasets (Project Achilles and GDSC), again out-performing clinically used genetic markers. Measurement of this minimal set of three protein biomarkers could thus inform treatment, and predict mechanisms of drug resistance in HER2+ cancers. More generally, our results show a single oncogenic transformation can have differing effects on cell signaling and growth, contingent upon the molecular and cellular context.

## Author Summary

Biomarkers capable of accurately predicting patient responses to alternate therapies are critical to realizing the vision of precision medicine. Identifying such biomarkers is, however, challenging due to the inherent complexity of biological networks. Here we sought to identify molecular features that predict how a genetically defined subset of cancers (HER2+) differentially depend on two oncogenic signaling pathways, the PI3K/AKT and MAPK/ERK cascades. We find that combined measurement of three non-intuitive proteins (EGFR, ERBB3, and CDKN1B) accurately predicts cellular dependence on these signaling pathways, and responsiveness to drugs targeting their constituents. Notably, this three-biomarker model outperformed both biological intuition (phosho-AKT and phospho-ERK) and current clinical practice (PIK3CA mutations). More broadly, this exemplifies how the functional consequences of a single oncogenic driver (HER2) can depend upon molecular and cellular context. Expression of these markers also varies by indication, such that breast cancers are biased toward PI3K-dependnece, while non-breast indications (lung, ovarian, and gastric) are particularly MAPK-dependent, and thus may respond differently to therapeutic strategies developed for breast cancer. Together, we believe that our results will aid the design of novel, stratified treatment strategies for HER2+ disease.

## Introduction

The elevated rate of proliferation and apoptotic-resistance characteristic of cancer cells depends on the activation of oncogenic signaling pathways. Such oncogenic pathway dependence creates molecular vulnerabilities, which can be exploited by targeted therapies. The effectiveness of such drugs however requires prospectively identifying which specific pathway(s) among many possibilities a given tumor is dependent on. This is a non-trivial task given the molecular and genetic heterogeneity of the disease, and the complexity of cell signaling networks. As a result, the majority of patients treated with targeted anti-cancer drugs fail to respond, and those that do often develop resistance over time [1].

The receptor tyrosine kinase HER2 is prototypic of oncogene addiction and a target for personalized anti-cancer therapy [2]. Overexpression of the receptor *via* amplification of the gene *ERBB2* results in ligand-independent homo-dimerization and constitutive signaling [3] primarily through the phosphoinositide 3-kinase (PI3K) cascade [4,5]. The monoclonal antibody trastuzumab (Herceptin; Genentech) is standard of care therapy for HER2^+^ disease. While its use has significantly reduced mortality from HER2+ breast cancer since approval in 1998 [6], many patients do not respond to treatment, particularly those with metastatic disease [7]. While subsequent HER2-targeted agents lapatinib, pertuzumab, and ado-trastuzumab-emtansine (T-DM1) have improved survival as components of combination regimens, patients still progress on these therapies [8]. Mutational activation of the PI3K pathway (*via PIK3CA* point mutations or *PTEN* deletions) is known to mediate resistance to HER2-targeted therapies in both pre-clinical models and through retrospective analysis of clinical data [9]. Consequently, many small molecules targeting components of the PI3K cascades, including PI3K, AKT, and mTOR inhibitors, are currently undergoing clinical trials in combination with HER2 therapy [10].

The mitogen activated protein kinase (MAPK) signaling cascade is another pathway hyper-activated in a large number of cancers, and many small molecule inhibitors targeting its pathway components such as BRAF [11] and MEK [12] are approved or in clinical development. While critical for transducing signals emanating from oncogenes such as *KRAS* [13] and other receptor tyrosine kinases including ErbB-family receptors [14], the pathway is not known to play a critical role in HER2-amplified cancers. On the other hand, the dual inhibition of PI3K and MAPK cascades can result in synergistic effects on cell proliferation and apoptosis in multiple cancer models [15], including HER2^+^ breast cancer [16,17], suggesting a potential role of MAPK signaling in the growth and survival of HER2+ cancers.

Many combinations of targeted therapies are currently undergoing clinical evaluation for treating trastuzumab-refractory HER2+ disease, including small molecule inhibitors of HER2, histone deacetylases (HDAC), heat shock proteins (HSP), insulin-line growth factor-1 receptor (IGF-1R), and the HER2 binding partner ERBB3 [8]. However, the molecular and genetic determinants of sensitivity to these agents, let alone their combinations, remain largely obscure. Rational strategies to functionally classify tumors by dependence on oncogenic signaling pathways using minimal sets of biomarkers would thus be highly valuable in designing improved treatment strategies. The goal of this study was to characterize the dependence of HER2+ cancers on two such pathways, the canonical PI3K and MAPK cascades. Further, we explored whether such dependence can be predicted from phenotypic, proteomic, or genomic biomarkers that could ultimately be used to stratify patients and inform treatment strategies.

## Results

### PI3K *vs*. MAPK Pathway Bias varies across HER2+ cells and is affected by HRG stimulation

Amplified HER2 is known to signal predominantly through the PI3K/AKT pathway in breast cancers [4]. However, it is unclear whether different indications with this genomic alteration are wired similarly downstream of the receptor. Also, it is well established that the HER3 ligand heregulin (HRG) stimulates PI3K signaling through induction of HER2/HER3 hetero-dimerization [14]. Yet the degree to which this ligand affects MAPK signaling downstream of the ErbB receptors in different cellular contexts is unclear. To answer these questions, we examined whether dependence on the PI3K and MAPK signaling cascades varies across HER2+ cancers, both in the presence and absence of heregulin. Specifically, a panel of 18 HER2+, but otherwise diverse cell lines was assembled, including breast, lung, gastric/esophageal, and ovarian cancer models. To characterize pathway dependence, each cell line was treated with a full 5x6 dose combination matrix of AKT and MEK inhibitors MK-2206 and GSK-1120212 (trametinib). *In vitro* cell proliferation was then quantified *via* video microscopy over 96 hours. All cell lines tested displayed some sensitivity to at least one of the inhibitors used.

To characterize the shapes of these response surfaces, quantitative logic-based models of cell growth kinetics were parameterized for each cell line. These phenomenological models characterize the balance of cell proliferation vs. cell death as functions of drug concentration (and by extension, pathway dependence) using combinations of quantitative logic gates. While nine alternate model variations were assessed (**S1 Table**), a logical OR-Gate regulating cell survival as a function of active (phosphorylated) AKT and ERK was found to perform optimally across the panel (**S1–S3 Figs**, **S4 Fig**). With only six parameters, it has the additional benefit of easy interpretation for comparison between cells. The six model parameters characterizing each cell consist of the maximal proliferation rate and cell death rates (*µ*_*MAX*_, *δ*_*MAX*_), EC_50_ and Hill coefficients characterizing inhibitor dose-responses (*τ*, *k*), and empirical weights toward PI3K and MAPK dependence (*w*_*AKT*_, *w*_*ERK*_) (see Materials and Methods and **S2 Table**). To develop a single metric of relative PI3K vs. MAPK pathway dependence, we define Pathway Bias as the normalized difference of the weighting parameters, where a value of 1 signifies complete PI3K-dependence (*w*_*AKT*_ >> *w*_*ERK*_), 0 dual-dependence (*w*_*AKT*_ ~ *w*_*ERK*_), and -1 complete MAPK-dependence (*w*_*AKT*_ << *w*_*ERK*_).

**Fig 1:**
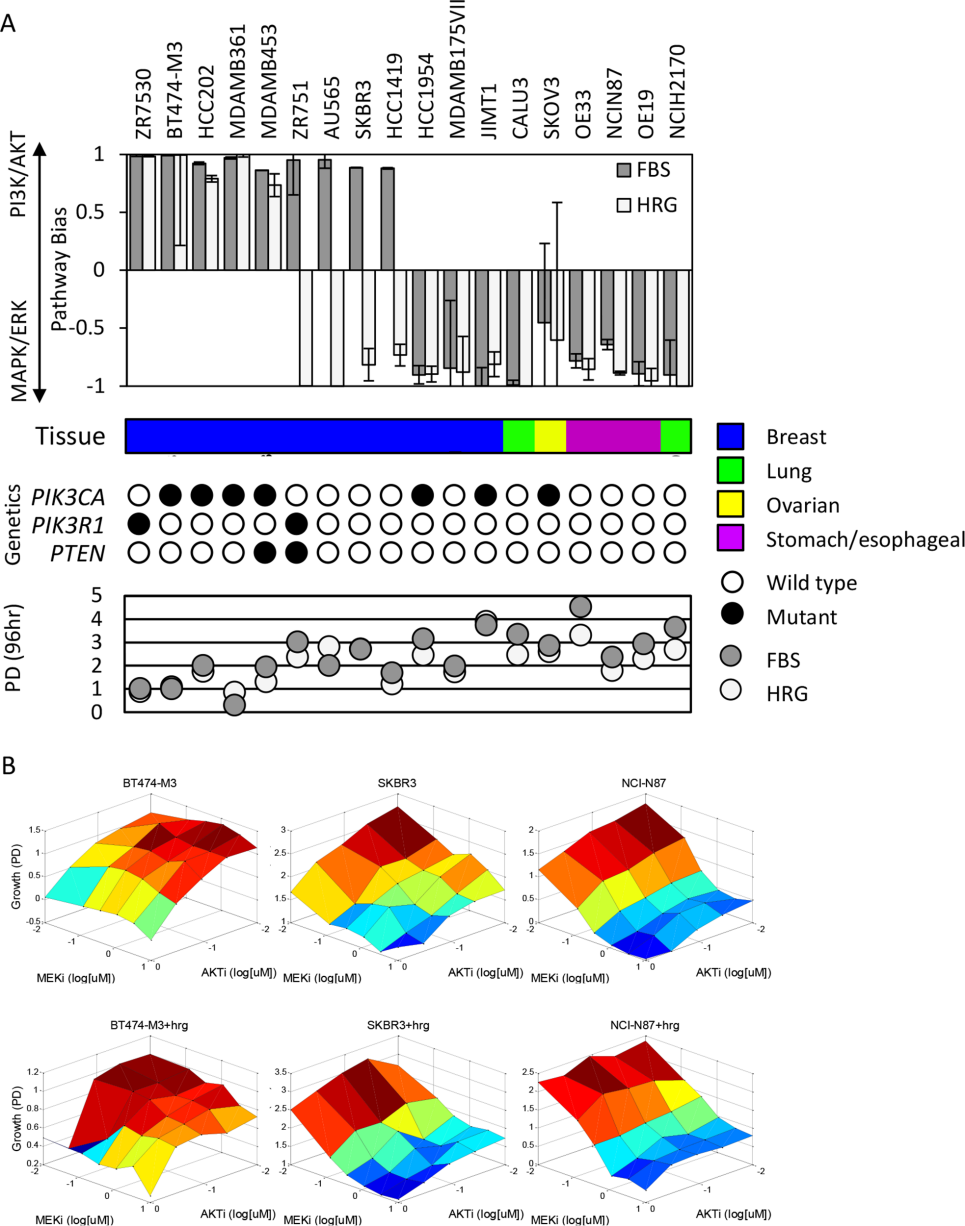
HER2+ cancer models vary by their dependence on PI3K *vs*. MAPK pathways and cellular properties. **(A)** Relative PI3K/AKT vs. MAPK/ERK dependence (Pathway Bias) across 18 HER2+ cell lines in the presence and absence of exogenous Heregulin (HRG) stimulation. Error bars represent 95% CI. Tissue, genetic status of PI3K-pathway components, and *in vitro* proliferation rates (96 hr population doublings; PD) are indicated below for each cell line. **(B)** Representative surface responses to AKT and MEK inhibitor combinations in the absence (top) and presence (below) of exogenous HRG for PI3K-dependent, MAPK-dependent, and switching class cells.

As shown in **Fig 1A**, 5/18 cell lines are classified as PI3K-dependent, 9/18 as MAPK-dependent, and unexpectedly 4/18 switch from PI3K to MAPK-dependence upon HRG stimulation. Error bars shown represent 95% confidence intervals (2 standard deviations) from 100 parameter estimation runs. The *Bias* estimates are very well constrained, with a median coefficient of variation of 3.7% (**S5 Fig,** part B). Only the SKOV3 cells would be considered undetermined (95% CI cross the axis), a result of the uniquely profound synergistic response of these cells to dual pathway inhibition (**S2 Fig**). Our models implicitly assume the PI3K/AKT and MAPK pathways function independently (i.e. no “cross-talk”), a shortcoming revealed by this specific case. Our primary motivation with the models were for data compression; reducing a 30-point response surface to three intuitive parameters (rates of cell proliferation, cell death, and pathway bias). While complexities could be added to the current models to better capture this phenomenon with SKOV3 cells, this is beyond our primary motivation. Representative surface responses for each class are shown below in **Fig 1B**. Heregulin stimulation reduced the sensitivity of all cells to AKT inhibition, and correspondingly increased relative sensitivity to MEK inhibition, though the effect was much more pronounced in the switching class (**S5 Fig**). Heregulin is known to desensitize cells to PI3K inhibitors [18], however the converse increased relative sensitivity to MEK inhibition was unexpected.

Overlaying information on basal proliferation, mutational status of three PI3K pathway key genes, and tissue source reveals some interesting patterns. First, consistent with the canonical classification of the MAPK pathway as mitogenic [14], we observed that proliferation rate (population doubling; PD) correlates with MAPK-dependence. Mutational status of the PI3K pathway, while correlated with PI3K-dependence, is not a predictive classifier. That is, while PI3K-biased cells are enriched for *PIK3CA*, *PIK3R1*, and *PTEN* mutations, some MAPK-dependent cells harbor *PIK3CA* mutations. These genetic metrics alone (HER2 amplification and PI3K pathway mutations) are thus insufficient for determining dependence of the tumor cells on PI3K vs. MAPK signaling. Most interestingly, our results show that, whereas breast cancers cover all three functional classes, all of the non-breast indications are MAPK-dependent. These are clinically significant findings, suggesting that current use of PI3K/AKT inhibitors in either unselected HER2+ cancer patients, or based on *PIK3CA* and *PTEN* mutations may be sub-optimal [8], and some HER2+ patients may benefit from treatment MEK inhibitors.

### Expression patterns of EGFR, ERBB3, and CDKN1B proteins are predictive of Pathway Bias

**Fig 2:**
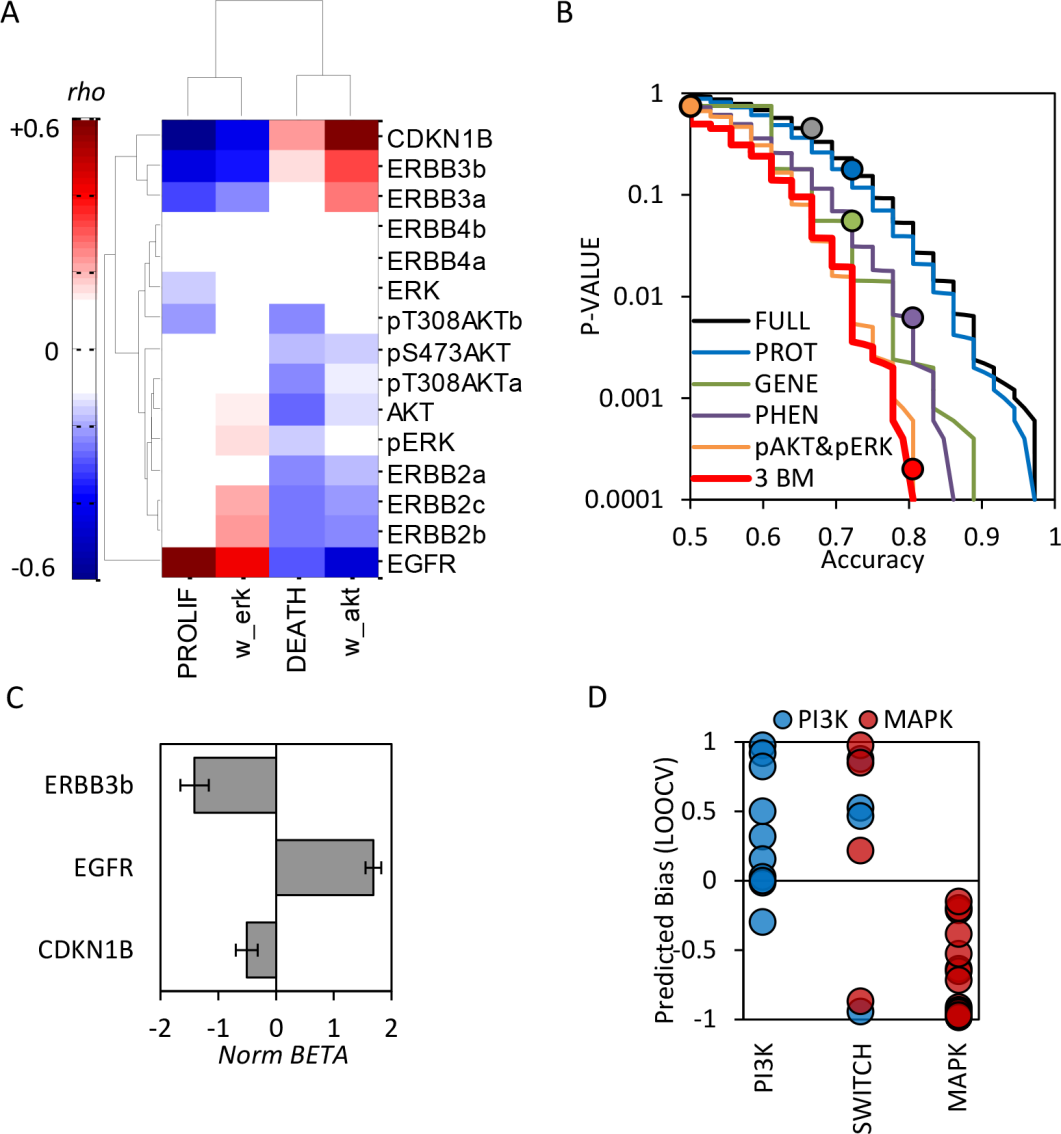
Multivariate protein biomarkers predict pathway dependence. **(A)** Rank correlation coefficients between protein expression and four model parameters, hierarchically clustered by Pearson correlations. **(B)** Accuracy of Pathway Bias predictions from Logistic regression models built on all input features (FULL), all protein measurements (PROT), PI3K pathway genetic status (GENE), cellular phenotype (tissue source and proliferation rate; PHEN), or 3 protein biomarkers EGFR, ERBB3, and CDKN1B (3BM). Results (filled circles) are overlaid on cumulative distributions from 10,000 randomized models (lines), thus relating predictive accuracies to statistical significance. **(C)** Normalized regression coefficients (BETA × median signal) for the three protein biomarker model. **(D)** Model-predicted Pathway Bias using the three protein biomarkers, separated by PI3K, SWITCH, and MAPK sub-groups.

To better define the HER2+ patient sub-populations that could respond to PI3K/AKT or MEK inhibitors, we sought to identify molecular features of the cells that are predictive of dependency on the PI3K/AKT and MAPK signaling. Applying a targeted proteomics approach, the same panel of cell lines was profiled for ErbB receptor expression, total and phosphorylated forms of ERK and AKT, and the cell cycle regulator CDKN1B (P27) using quantitative Luminex assays (**S6 Fig;** raw data provided in **S1 Data**). The relationships between protein expression and cellular functional properties were then analyzed by computing Spearman’s rank correlation coefficients between protein measurements and the characteristic model parameters across the panel of cell lines (**Fig 2A**). Some of the protein species were quantified with more than one detection antibody (annotated a, b, c) as a quality control check. The effect of heregulin stimulation was accounted for solely by its effect on cell signaling, as each cell line +/- heregulin was treated as two independent samples. For our analysis of the proteomic and cellular response data, we treated the same cell line +/- heregulin treatment as independent samples, thus producing 36 (18x2) samples. Functional relationships are revealed from these correlations; highly proliferative cells express increased levels of EGFR and are MAPK-signaling dependent, while slowly proliferating cells have higher levels of ERBB3, CDKN1B (p27), and are PI3K-dependent. This is consistent with the canonical association of PI3K and MAPK pathways with regulating cell survival and proliferation, respectively [14], but to our knowledge the first instance of this functional partition revealed in a purely data-driven manner. To further explore this relationship, we created a tenth model variant (M10) which explicitly encodes proliferation and cell survival as separately regulated by MAPK and PI3K/AKT signaling, respectively (see Methods). The model parameters were estimated from the surface response data as with the previous nine, and parameter estimates, simulations, and goodness of fit metrics (MSE and AIC values) are shown in **S1 Data** for all. In comparison to the chosen model (M4), this variant produced a better fit to the data in the majority (21/36) of samples, with average MSE slightly reduced by 0.5%. This further supports the observed relationship between cell proliferation and MAPK signaling, and cell survival with PI3K/AKT signaling. It is notable that neither the phosphorylated or total amounts of AKT or ERK proteins correlated with pathway dependence, confounding our naïve expectations. This non-intuitive finding is nevertheless consistent with previous studies examining biomarkers of PI3K/AKT and MEK inhibitor sensitivity in different cancer models [19–21].

To assess whether these molecular correlations were predictive, logistic regression models were parameterized to classify cells as PI3K vs. MAPK-dependent using different sets of input features (protein expression, PI3K pathway mutational status, the phenotypic properties of proliferation rate and tissue origin [breast vs. non-breast], or all features combined). Models were evaluated for predictive accuracy via leave-one-out cross validation (LOOCV), and compared against 10,000 random permutations to assess statistical significance (**Fig 2B**). Consistent with the correlation analyses, the most intuitive biomarkers, pAKT and pERK, were in fact no better predictors of Pathway Bias than random chance (Accuracy = 50%, *P* = 0.75). Accuracy of the “full” model (containing all molecular, genetic, and phenotypic features), the protein-based model, and the genetic model were quite poor (67%, 72%, and 72% corresponding to *P*-values of 0.45, 0.18, and 0.055). In contrast, model predictions based solely on “phenotype” (tissue and proliferation rate) were quite accurate (81%, *P* = 0.006).

To assess whether a subset of the protein measurements could provide a molecular explanation for this result, a model was built using the 3 proteins best correlated with pathway bias: EGFR, ERBB3, and CDKN1B. The accuracy of this 3-biomarker model matched the phenotype-based predictions (81%, *P* = 0.0002), and was statistically superior to all alternatives assessed (**S7 Fig**). Combining the 3 protein biomarkers and phenotypic features did not improve accuracy, demonstrating redundancy between these measurements. That is, the observed association between tissue, proliferation rate, and pathway dependence can be explained solely by differential expression of these three proteins. Relative importance of the three protein features can be inferred from the normalized regression coefficients (**Fig 2C**; raw data provided in **S3 Table** and **S4 Table**), in order of descending importance EGFR, ERBB3, and CDKN1B.

The ability of heregulin to shift pathway dependence from PI3K to MAPK is an unexpected observation, given that this growth factor is commonly associated with PI3K signaling. Consistent with this established role, pAKT (pS473 and pT308) was induced in the majority of cells treated with the ligand (**S8 Fig**). And while pAKT induction was greater in the PI3K-depdendent cells, this nor any other single protein change consistently correlated with pathway dependence switching. Including heregulin treatment as an additional discrete feature (1/0) in addition to the three protein biomarkers did not improve model accuracy (78%, *P* = 0.001). The context-dependent *Bias* of the four cell lines which switch dependence was poorly predicted (**Fig 2D**). In fact, the majority of the error in model (4 of 7 misclassifications) is attributable to its inability to predict the switching behavior as the AU565, HCC419, and ZR751 cell lines are classified as PI3K-depdendent, and SKBR3 as MAPK-dependent, regardless of heregulin. The 3 protein biomarkers are thus able to accurately predict intrinsic dependence on PI3K/AKT vs. MAPK signaling. However, the shift in dependence induced by heregulin stimulation may occur through alternative mechanisms not accounted for in our panel of protein measurements.

### Biomarker expression patterns in clinical tumor samples

**Fig 3:**
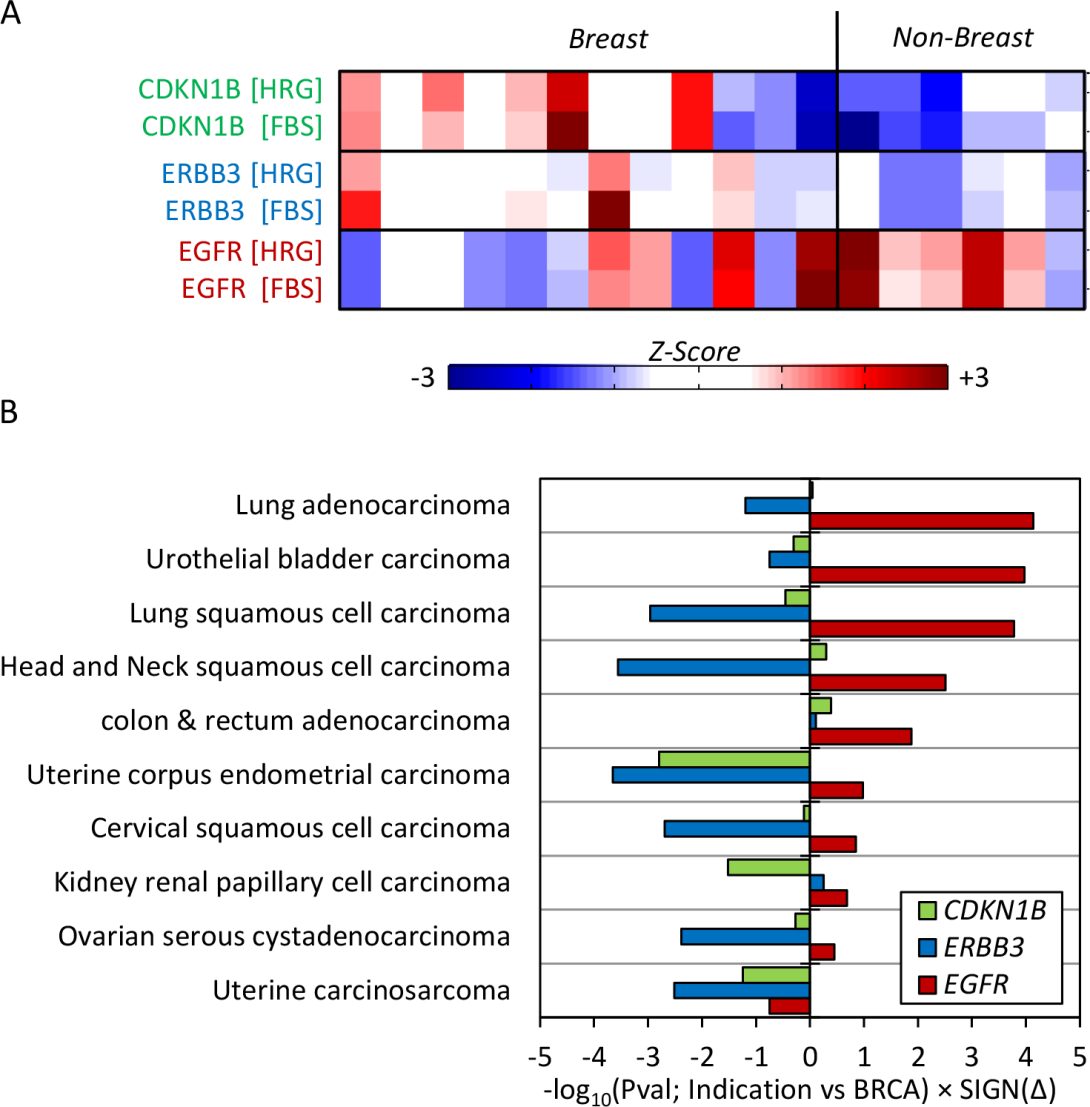
TCGA Analysis of biomarker expression in HER2+ cancers. (A) Protein expression of CDKN1B, ERBB3, and EGFR across the 18 cell lines +/- heregulin treatments, scaled by row and organized as in Fig 1, categorized as breast vs. non-breast. (B) mRNA Expression of the same three biomarkers (*EGFR*, *ERBB3*, and *CDKN1B*) in 10 HER2+ cancer indications as compared to breast cancer, expressed as signed *P*-values (rank-sum test).

We observed that all non-breast cell lines are MAPK-dependent, and this is explained by expression of the three protein biomarkers (**Fig 3A**). Thus, if cell lines are representative of the derivative disease, one would expect to see higher levels of EGFR and lower levels of ERBB3 and CDKN1B in non-breast indications vs. breast tumors. To test this hypothesis, we next queried available clinical gene expression data from The Cancer Genome Atlas (TCGA) for expression of EGFR, ERBB3, and CDKN1B by indication. RNAseq profiling data (V2 RSEM) was extracted for all indications available, and classified as HER2+ vs. HER2- sub-classes for analyses based on *ERBB2* gene expression (**S9 Fig**). Consistent with molecular profiles of the cell lines, in all 10 indications with significant numbers of HER2+ samples, *EGFR* expression was relatively higher and/or *ERBB3* and *CDKN1B* lower in in comparison to breast cancers (**Fig 3B**), and these patterns hold for both the HER2+ and HER2- subsets. The expression patterns observed in our panel of immortalized cell lines are thus consistent with their derivative indications, suggesting that non-breast HER2+ cancers are likely to be dependent on MAPK rather than PI3K signaling. We speculate this may arise though differential hetero-dimerization, with ERBB2-EGFR complexes preferentially activating MAPK signaling, and ERBB2-ERBB3 PI3K/AKT signaling.

### EGFR, ERBB3, and CDKN1B expression predict sensitivity to genetic knockdown and chemical inhibition of PI3K and MAPK pathways in independent datasets

To validate the functional utility of the three protein biomarkers (EGFR, ERBB3, and CDKN1B), we assessed whether they could predict pathway dependence using data from independent experiments.

**Fig 4:**
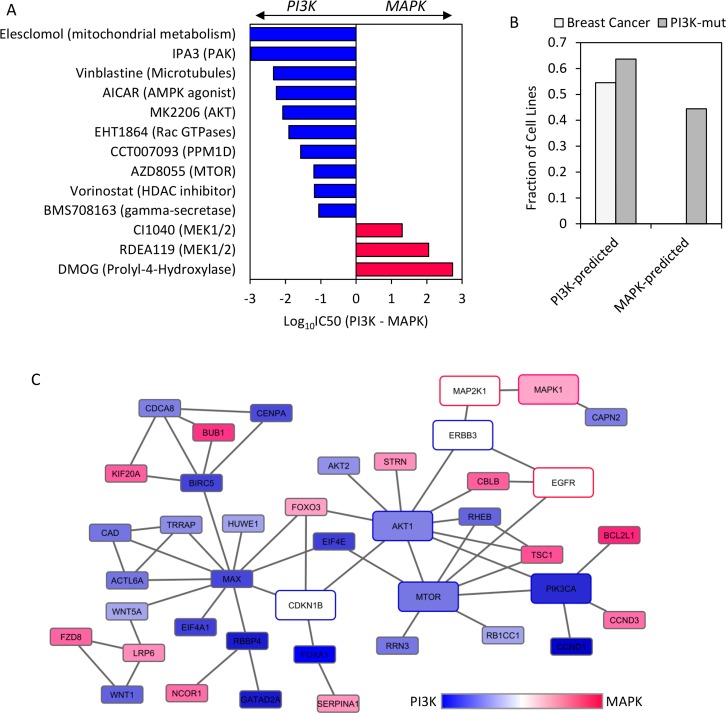
Differential signaling networks, cellular features, and drug responsiveness between PI3K and MAPK-biomarker stratified HER2+ cancer cell lines. **(A)** Differences in drug sensitivity (log_10_(IC_50_)) between PI3K vs. MAPK biomarker-enriched cell lines, based on protein measurements of EGFR and ERBB3, and filtered by Rank-sum *P*-value < 0.1. Bars are color-coded accordingly by predicted PI3K (blue) vs. MAPK (red) dependence. **(B)** Frequency of PI3K mutations (*PIK3CA*, *PIK3R1*,and *PTEN*) and tissue source (Breast vs. Non-breast cancers) in the two groups of cell lines. **(C)** Genes identified as differentially sensitive between the biomarker-defined cell line subsets (*EGFR*^HI^*ERBB3*^LO^*CDKNIB*^LO^ vs. *EGFR*^LO^*ERBB3*^HI^*CDKNIB*^HI^) were mapped onto oncogenic signaling networks in NCI-PID and color-coded by differential association.

First, we sought to determine whether protein expression of EGFR, ERBB3 and CDKN1B, could be used to predict differential sensitivity to anti-cancer drugs in HER2+ cancers. An ELISA-based protein profiling dataset across 90 cancer cell lines [22] was intersected with the Genomics of Drug Sensitivity in Cancer database (GDSC; 714 cell lines screened for sensitivity to 138 cancer drugs [23]). While CDKN1B measurements were not available, the relevance of EGFR, ERBB3, pAKT, and pERK as predictive biomarkers were evaluated by focusing on the eight PI3K/AKT/MTOR inhibitors and four MEK inhibitors in the GDSC database (**S6 Table)**. Within the HER2hi population (the 67th percentile, corresponding to 22 intersecting cell lines), differential sensitivity (IC50s) to each of the agents was evaluated between the biomarker-high vs. low group, defined by median cuts and statistical threshold of *P* < 0.1 (rank-sum test). Consistent with the logistic model, EGFR was the best single marker, identifying 1 of 8 PI3K/AKT/MTOR and 2 of 4 MEK inhibitors. While neither pAKT nor pERK expression predicted differential sensitivity to any of the drugs (**S6 Table**), examining combinations of the biomarkers, comparing EGFR^lo^ERBB3^hi^ (PI3K-bias) vs. EGFR^hi^ (MAPK-bias) yielded 2 of 8 PI3K/AKT/MTOR and 2 of 4 MEK inhibitors as differentially sensitive between the two groups (**Fig 4A**).

Based on this EGFR^lo^ERBB3^hi^ (PI3K-bias) vs. EGFR^hi^ (MAPK-bias) classification scheme, thirteen drugs were found to display differential sensitivities between the groups (**Fig 4A**). This includes the AKT inhibitor used in our studies (MK2206), and the MEK inhibitors CI1040 and RDEA119. The PI3K-predicted subset was also increasingly sensitive to AZD8055, an agent targeting MTOR, a canonical downstream effector of this pathway.

Examining properties of the cell lines in each cohort, it is notable that the PI3K-predicted subset was relatively enriched in breast cancers (55% vs. 0%), and PI3K pathway mutations (*PIK3CA*, *PTEN*, or *PIK3R1*; 64% vs. 44%) as compared to the MAPK-predicted subset (**Fig 4B**), consistent with the characteristics of our internal 18 cell line panel.

It was however unclear as to why only a subset of the AKT and MEK inhibitors came up in this analysis. To explore the reason for this discrepancy, we examined patterns of sensitivity to the drugs across all 714 cell lines in the database by computing pair-wise Spearman correlation coefficients between their IC_50_ values. As depicted in the correlation matrix in **S11 Fig**, sensitivity to inhibitors of the same pathway, and even the same target across cell lines are often poorly correlated. For example, correlation coefficients between the AKT inhibitor we employed (MK-2206) and the two other AKT inhibitors in the dataset (AKT Inhibitor VIII and A-443654) are 0.22 and 0.08. Correlations between sensitivity to MK-2206 and the four PI3K inhibitors vary between 0.23 (AZD6482) and 0.55 (GDC0941), and the four MTOR inhibitors from 0.16 (Rapamycin) to 0.42 (AZD8055). Correlations between the four MEK inhibitors are significantly better (0.61 to 0.75) but still not nearly as tight as would be expected for inhibitors of the same target. These discrepancies may be attributable to different mechanisms of action, off-target specificities between the alternate inhibitors, or other technical issues. Regardless of the underlying reason, this could explain why our biomarker stratification scheme only identified a subset of the MEK and PI3K/AKT/MTOR inhibitors.

We next sought to validate our predictions using functional genomics data. Here, we utilized two cancer cell line data repositories; mRNA expression profiles from the Cell Line Encyclopedia (CCLE; [24]), and functional genomics data from Project Achilles, which catalogues vulnerabilities of cancer cell lines to shRNA or Cas9/sgRNA-mediated gene silencing [25]. We first assessed whether mRNA expression of the biomarkers could substitute for protein. EGFR, ERBB2, and CDKN1B gene expression correlated well with protein levels across the 18 HER2+ cell line panel (Spearman ρ = 0.84, 0.67, 0.77) but ERBB3 less so (Spearman ρ = 0.50; **S10 Fig,** part A). The poorer correlation between ERBB3 mRNA transcript and protein may be attributable to the multiple feedback circuits regulating expression of this receptor [26]. Also consistent with protein expression patterns, CDKN1B transcript expression is positively correlated with ERBB3 and anti-correlated with EGFR (Spearman ρ = 0.27 and -0.23 respectively; **S10 Fig,** part A).

Using the CCLE mRNA expression data, we classified cell lines in the dataset as HER2+ and HER2- based on the 80th percentile of ERBB2 expression, and examined differences in sensitivities to gene knockdowns between cell lines based on expression of EGFR, ERBB3, and CDKN1B mRNAs. Among the 43 HER2+ cell lines found in the Achilles portal, 11 were predicted to be PI3K-dependent (EGFR^lo^ERBB3^hi^CDKN1B^hi^) and 9 to be MAPK-dependent (EGFR^hi^ERBB3^lo^CDKN1B^lo^) based on median cuts of the 3 biomarkers. Of the 5711 genes tested for growth dependence, 781 showed differential sensitivity between the two sets of cell lines (*P* < 0.05, rank-sum test). This is almost 3-fold more than expected by chance, suggesting real biological differences between the biomarker-defined subsets. The PI3K-predicted cells were significantly more sensitive towards silencing of three canonical PI3K/AKT signaling nodes, PIK3CA, AKT1 and MTOR. Within the MAPK-predicted set, while MEK1 (MAP2K1) did not come up as a differentially sensitive target, the main MAPK effector ERK2 (MAPK1) did. A full list of the genes and associated statistics is provided in **S1 Data**, both for the combined three biomarker results, and each biomarker in isolation.

To evaluate the relative utility of this three-gene classifier, we performed the same analysis using *PIK3CA* mutation status as a predictor of PI3K pathway dependence. This is the most widely used clinical biomarker associated with the use of PI3K/AKT inhibitors, and thus could be considered the gold standard comparator, despite being a poor predictor of clinical activity in actuality [27]. *PIK3CA*-mutant cells are indeed significantly more sensitive to knock-down of *PIK3CA* itself, as well as *MTOR* within the HER2+ population. However, *AKT1* dependence was not associated with *PIK3CA* mutants, nor was either of the two MAPK targets (*MAP2K1* and *MAPK1*) associated with the *PIK3CA*-wildtype cells (**Table 1**). Applying the same analyses to HER2- cells reveals that the relationship between expression of the three genes and pathway dependence is specific to HER2+ cancers; only *AKT1* comes up as differentially sensitive target using the three biomarker combination, while *PIK3CA*, *MTOR* and *MAPK1* do not, as in the HER2+ population. The predictive utility of *PIK3CA* mutations however appears independent of HER2 status, as the same target (*PIK3CA*) comes up in both HER2+ and HER2- groups. The identification of 4/5 canonical gene targets (PIK3CA, AKT1, MTOR, MAP2K1, and MAPK1) as differentially sensitive using the three biomarker enrichment strategy in HER2+ cells is highly unlikely to be due to chance alone (*P* = 1.5 × 10^−3^, Hyper-Geometric test), much more so than the 2/5 targets uncovered using *PIK3CA* mutational status. The three-biomarker set thus appears a better differentiator of PI3K/AKT vs. MAPK/ERK pathway dependence in HER2+ cells as compared to commonly used genetic marker *PIK3CA*.

**Table 1:** Canonical PI3K/AKT and MAPK/ERK signaling nodes and their statistical association with biomarker-defined cell line subsets represented in Project Achilles.

	3BM (HER2+)	3BM (HER2-)	PIK3CA (HER2+)	PIK3CA (HER2-)
**Rank-Sum**	P-VAL	PVAL	PVAL	PVAL
*AKT1*	3.9×10^−3^	6.9×10^−3^	5.2×10^−1^	6.6×10^−1^
*PIK3CA*	9.8×10^−3^	1.8×10^−1^	2.8×10^−2^	6.1×10^−3^
*MTOR*	3.9×10^−3^	1.9×10^−1^	4.6×10^−2^	9.1×10^−2^
*MAP2K1*	4.0×10^−1^	4.2×10^−1^	6.4×10^−1^	3.9×10^−2^
*MAPK1*	4.8×10^−2^	8.8×10^−1^	6.6×10^−1^	3.8×10^−1^
N (total Genes)	5711	5711	5711	5711
n (*P* < 0.05)	781	1495	344	322
r (hits/5)	4	1	2	2
**Hyper-geometric**				
P-VAL	1.5x10^-3^	7.8x10^-1^	3.2x10^-2^	2.8x10^-2^

To interrogate the molecular mechanisms underlying this relationship, we mapped the differentially sensitive genes on to the NCI pathway interaction database (NCI-PID), a curated resource of cancer-associated signaling pathways [28]. To limit the network size and enrich for biologically meaningful components, we further filtered for genes with median differential effects (ATARiS scores [29]) > 0.75 and with at least one interaction annotated in the NCI database, and included EGFR, ERBB3, CDKN1B, and MAP2K1. The resulting network, consisting of 41 nodes and 56 edges, is represented in **Fig 4C**. Besides direct protein-protein interactions, an edge in the network could represent transcriptional and translational regulation, as well as a macroprocess whose internal composition is not included [28]. Many core components of PI3K/AKT signaling and downstream effectors are connected (i.e. E1F4E, RHEB, FOXA1, MAX, CCND1, AKT2, TSC1) in a giant component associated with PI3K-pathway dependence, while the MAPK-predicted genes are more diffuse, and cover diverse signaling pathways and mechanisms (such as components of the WNT signaling pathway). The three biomarkers (EGFR, ERBB3, and CDKN1B) are connected with each other, both directly and through the intermediary network hub AKT1. These connections suggest that the three biomarkers are functionally linked to AKT and MAPK signaling, and their relative expression levels thus could induce differential dependence on the two signaling cascades.

Together, these results support our initial finding that that EGFR, ERBB3 and CDKN1B expression predict differential dependence on PI3K/AKT and MAPK signaling in HER2+ cancer cells.

## Discussion

Our finding that many HER2+ cancer cell lines are dependent on MAPK signaling contrasts with canonical view of HER2 signaling predominantly through the PI3K pathway [4,5]. We believe this novelty is due to our profiling of a larger and more diverse panel of HER2+ cell lines than any previous study to our knowledge, and the fact that MEK inhibitors are typically not examined in these cells as a result of this established dogma. The finding is however not completely unprecedented; a recent study describing the construction of Boolean network models using proteomic data from HER2+ cell lines revealed that the cell lines varied in their intrinsic bias toward PI3K vs. MAPK signaling [30]. If results translate beyond in vitro cell culture, this finding has implications for the design of treatment strategies in HER2+ cancers, as multiple PI3K/AKT/MTOR inhibitors are being tested in HER2+ cancers, and MEK inhibitors are being tested in a variety of other tumor types [12]. Combined measurement of these three proteins in tumor biopsies could thus inform the use of PI3K/AKT or MEK inhibitor treatments. It is worth noting that mutations in any of these three genes may affect their predictive utility in this context, however this rarely occurs in HER2+ cancers (less than 10% harbor mutations in *EGFR*, *ERBB3*, or *CDKN1B* [31]). Our results predict that MAPK pathway-activating mutations (such as *KRAS*^G12V^) may be genetic mechanisms of resistance to HER2-direted therapy in indications outside of breast cancer, with higher EGFR and lower ERBB3 and CDKN1B expression [32]. While clinical data supporting this prediction are lacking, mechanistic model simulations are consistent with the role of *KRAS* mutations as dominant mechanisms of resistance in MAPK-dependent HER2+ cancers [33].

As with all molecularly targeted agents, predictive biomarkers are needed to realize the utility of PI3K/AKT and MEK inhibitors. Our results highlight the difficulty in identifying such predictive markers, as the most intuitive protein (pAKT and pERK), and genetic (*PIK3CA*) candidates turned out to be largely uninformative and surpassed by a fairly non-intuitive multivariate classifier. These findings are consistent with clinical experience to date with PI3K/AKT/MTOR inhibitors [34] and MEK inhibitors [12], in that mechanistically intuitive genetic markers have proven poor predictors of activity. Results from large cell line-based functional genomics projects [35,36] more broadly support this finding. Despite substantial efforts to find robust genetic predictors of drug sensitivity, these have proven largely disappointing [37]. We believe the root of this challenge lies in two related sources. First, cellular dependence on a given signaling pathway may arise through multiple mechanisms, such as expression patterns of regulatory ligands, receptors and downstream effectors, in addition to mutations in core signaling nodes. Predictors of sensitivity to pathway targeted inhibitors are thus expected to be necessarily multivariate, and often non-genetic, which would favor the use of proteomic technologies for predictive biomarker discovery [38]. Second, the very properties of oncogenic signaling networks that confer robustness to therapeutic intervention (adaptive feedback circuits and redundancies) also obscure predictors of responsiveness to such interventions. In addition to our data, recent functional proteomic studies support these conclusions. Phospho-kinase expression has been proven a poor predictor of cellular sensitivity to inhibitors targeting those kinases and the cascades in which they are embedded, including pAKT and pERK in relation to PI3K/AKT and MEK inhibitors [20,39]. Similarly in line with our results, PI3K inhibitor sensitivity across a panel of breast cancer cell lines was predicted by responsiveness to the ERBB3 ligand heregulin much better than by pAKT expression level or *PIK3CA* mutations [19].

We speculate that differential HER2-heterodoimerization accounts for the association between ERBB3 vs. EGFR receptor expression and PI3K vs. MAPK pathway dependence. The EGFR cytoplasmic domain contains multiple binding sites for the adaptor proteins Growth-factor-Receptor-Bound 2 (GRB2) and Src-homology-2-containing (SHC) which activate the MAPK cascade, while ERBB3 has six binding sites for PI3K and only one SHC site [40]. Competition for binding to HER2 between the receptors could thus shift receptor complex formation to favor one pathway over the other. CDKN1B (p27) is likely a functional surrogate of pathway activity, rather than a causal regulator. As a negative regulator of cell cycle progression, its expression level may be indicative of the intensity of pro-proliferative MAPK signal flux. These results also demonstrate that the functional effect of an oncogene can be context-dependent. In this case, *ERBB2*-amplification can result in either PI3K or MAPK-signaling-dependent cell growth, depending on molecular context. Consistent with the “ERBB network theory” [14,41], signal output depends upon the composition of surface receptors and presence of extracellular ligands. Whether such context-dependent signaling effects are confined to the ErbB-family, or shared by other oncogenes is an open question. It is however clear that cancers harboring the same oncogenic driver can respond very differently to targeted inhibitors based on their tissue of origin, the most notable example being vemurafenib responses in *BRAF*^V600E^-mutant melanoma vs. colorectal cancers [42]. Context-dependent signaling differences may play a role in such cases.

The finding that PI3K vs. MAPK pathway dependence is not genetically hardwired into cells, and can be affected by exposure to the ligand heregulin was somewhat unexpected. However, there is precedent for observations of cellular plasticity with respect to reliance on oncogenic signals. Growth factor stimulation is known to mediate resistance to many kinase inhibitors through the activation of alternate but functionally redundant pathways [43,44]. PI3K/AKT inhibitors and MEK inhibitors themselves can also induce compensatory signaling though alternative pathways via the relief of negative feedback regulatory controls on cell surface receptors [16,45–47]. Many cancer cells are thus endowed with the capacity for using alternate pathways in response to environmental changes. While we have focused solely on the role of the ERBB3 ligand heregulin, other growth factors and cytokines may have similar effects. This is important to consider when interpreting biomarker-response relationships. If both molecular profiles and drug response patterns are fluid, such relationships would be amenable to shift under different experimental [48], and possibly pathophysiological conditions. In light of all the aforementioned challenges, it is perhaps unsurprising that despite all the resources and efforts committed date, a very limited number of predictive biomarkers have proven clinical utility [49]. Though counter-intuitive, strong relationships between inhibitor sensitivity and target expression appear to be the exception rather than the norm.

We were able to support our initial findings from the 18-cell line panel using both chemical genomic and functional genomic data from independent sources. Besides expected hits in the PI3K/AKT and MAPK/ERK signaling pathways, our three biomarker stratification scheme revealed differential sensitivity towards additional small molecules and shRNAs. It is likely that some of these are false positives. However, there appear to be mechanistic connections between the targets of these compounds and shRNAs, the three biomarkers, and PI3K/AKT and MAPK signaling. For example as shown in **Fig 4A**, cells predicted to be PI3K or MAPK dependent are also differentially sensitive towards an AMPK inhibitor (AICAR), consistent with known biological functions of PI3K/AKT signaling in metabolism [50]. In addition, histone deacetylase (HDAC) inhibitors are known to mediate at least part of their effects through suppression of PI3K/AKT signaling [51]. Elesclomol induces apoptosis through disruption of mitochondria metabolism and in cancer cells upregulates AKT signaling to promote survival [52]. BMS708163 targets presenillin1 as a NOTCH-sparing gamma-secretase inhibitor [53] and regulates 4ICD release upon NRG1 binding to ERBB4 [54]. While the exact mechanistic connections between these small molecules and EGFR/ERBB3 expression are unclear, these results are likely to be biological meaningful and not merely random. In addition, we performed a GO enrichment analysis [55,56]on biological processes for the genes from the Achilles’ analysis using genes tested in this dataset as background. We found that the shRNA targets are enriched for regulators of mRNA transcription, mRNA transport, translation, and mitotic progression. Consistently, the small molecules that we found in addition to direct inhibitors of PI3K/AKT/MTOR and MEK target components of the transcriptional, translational and cell cycle machinery. For example, vorinostat is known to cause abnormal mitosis through inhibition of HDAC [57]. Vinblastine has been shown to block mitosis through inhibiting microtubule dynamics indirectly or directly [58]. We also observed differential proliferation rates between AKT and MAPK-dependent cell lines experimentally (**Fig 1A**), consistent with differential sensitivity to knockdowns of cell cycle regulators. These results suggest that the hits from both analyses are likely to be mechanistically connected and biologically meaningful.

In summary, we have demonstrated that PI3K vs. MAPK pathway dependence varies across HER2+ cancer cells. This dependence varies by indication, and can be predicted using a set of three non-intuitive protein measurements. These results might help stratify HER2+ patients for treatment with targeted therapeutics. More generally, our findings reveal that oncogenic signaling can be context dependent. A single genetic transformation, in this case *ERBB2* amplification, can have differing effects on cell signaling and growth, contingent upon on the molecular and cellular background. Together, we believe that our results and approach will enable the design of more effective cancer treatment strategies for HER2+ cancer patients.

## Materials and Methods

### Cell lines and reagents

AU565, HCC1419, NCI-H2170, HCC202, HCC1954, NCI-N87, ZR75-1, SKOV3, ZR75-30, MDAMB175VII, CALU3, MDAMB453, MDAMB361, JIMT1, SKBR3 and HCC2218 cells were obtained from ATCC. OE19 and OE33 were obtained from ECCC; COLO-678 was obtained from DSMZ, and KYSE-410 from Sigma-Aldrich. BT-474-M3 cells (hereafter simply referred to as BT-474) were obtained from Hermes biosciences. All cell lines were maintained in RPMI supplemented with 10% FBS, penicillin, and streptomycin. GSK-1120212 and MK-2206 were purchased from Selleckchem. Recombinant human HRG-β1 (EGF domain) was from R&D Systems.

### In vitro cell growth assays: AKT & MEK inhibitor responses

Cells were seeded at 600 cells per 384-well plate in 4% FBS cell growth medium, stimulated (or not) with 2 nM HRG-b1 for 4 hours, and then treated with individual or combinations of the AKT and MEK inhibitors. Treatments consisted of 5x6 dose combination matrices covering a 3-fold dilution series from 1 μM (MK-2206) and 10μM (GSK-1120212). Cell confluency was then monitored over 5 days in culture by video microscopy (IncuCyte, Essen BioScience), and data normalized to density measured at initiation of treatment (**S1 Data**).

### Cellular protein lysate preparation

Cell lines were seeded at 7,500 cells per well in 384-well culture plates in RPMI containing 4% FBS. 48-hour post plating, cells were stimulated (or not) with 2 nM HRG-β1 for four hours. At harvest, cells were placed on ice, and 70 μl RIPA lysis buffer (Sigma-Aldrich) supplemented protease inhibitor and phosphatase inhibitor tablets (Roche) was added to each well. The plates were stored at -80°C until analysis. On the first day of protein profiling, the lysates were thawed at 4°C and centrifuged at 4000 rpm for 10 minutes. The supernatant was used for further analysis with multiplex Luminex protein assays as described below.

### Multiplex (Luminex) protein assays

Twenty micrograms of antibodies was conjugated to 100 μl (~1.25×10^6^ beads) of MagPlex beads (Luminex Corp.) according to the manufacturer’s instructions. Conjugated beads were then mixed and diluted 1000-fold in phosphate buffered saline (PBS)–1% bovine serum albumin (BSA) (Sigma). Diluted beads were transferred into 384-well assay plates (Corning) at 30 μl per well and then washed three times with PBS–1% BSA. Washed beads were incubated with 20 μl of total protein lysates overnight with shaking at 4°C. The beads were then washed with PBS–1% BSA. Detection antibodies (see S3 Table) were added and incubated at 4°C overnight with shaking. After washing with PBS–1% BSA, streptavidin-conjugated phycoerythrin (Invitrogen) was added at 2 μg/ml and incubated at room temperature for 30 min. Finally, the beads were washed with PBS–1% BSA, and data were acquired with a FlexMap3D instrument (Luminex Corp.) according to the manufacturer’s instructions. Raw signals were normalized by background subtraction to signals from control lysates prepared from non-human cells. Antibodies are listed in **S3 Table**, and background-subtracted data is provided in **S1 Data**.

### Logic-based models of cell growth regulation

Observed changes in cell density over time are determined by the balance of cell proliferation vs. death within the culture. Both cell proliferation and survival are regulated by PI3K/AKT and MAPK/ERK signaling cascades, which assuming an exponential growth can be expressed as:

$$\frac{dX}{dt}=\;\mu_{MAX}\cdot f_{1}(pAKT,pERK)-\;\delta_{MAX}\cdot f_{2}(pAKT,pERK)$$

Where *X* = number of cells (assumed proportional to surface area), *μ*_*MAX*_ = maximum rate of proliferation, *δ*_*MAX*_ = maximal rate of cell death, and *f*_*1*_ and *f*_*2*_ are functions integrating pAKT and pERK signaling.

We implemented a quantitative logic-based formalism [59] to describe changes in cell density as function of PI3K/AKT and MAPK/ERK pathway activation. AKT and MEK inhibitor concentrations (μM) were used as surrogates for pathway activities, assuming monotonic dose-response relationships. As the logic by which cells integrate and interpret these signals remains obscure, we initially assessed 9 alternate growth regulatory functions combining null (K), OR, and AND-type logic gates as proliferation and survival functions (*f*_*1*_ and *f*_*2*_):

K=1

$$OR=\;\frac{{\left(w_{akt}\cdot AKT+\;w_{erk}\cdot ERK\right)}^{k}}{\tau +\;{\left(w_{akt}\cdot AKT+\;w_{erk}\cdot ERK\right)}^{k}}$$

$$AND=\;\left(\frac{{AKT}^{k\_akt}}{\tau_{akt}+\;{AKT}^{k\_akt}}\right)\cdot \left(\frac{{ERK}^{k\_erk}}{\tau_{erk}+\;{ERK}^{k\_erk}}\right)$$

Parameters for each of the 9 models (**S1 Table**) were estimated for each cell line using a Particle Swarm Optimization algorithm [60] minimizing the mean squared error between experimental measurements (fold cell expansion over 96 hours) and model simulations. Relative model performance was assessed using the Akakie Information Criterion (AIC):

$$AIC=\;2\cdot P+N\cdot {\text{log}}_{10}(MSE)$$

Where *P* = number of parameters (2–10), *N* = number of experimental measurements (30), and *MSE* = mean squared error.

The fourth model structure assessed (M4), consisting of an OR-Gate regulating cell survival, was found to be optimal (lowest AIC) for the largest number of cell lines tested. The final formulation of the cell growth regulatory model used in subsequent analyses was thus:

$$\frac{dX}{dt}=\;\mu_{MAX}-\;\delta_{MAX}\left(\frac{{\left(w_{akt}\cdot AKTi+\;w_{erk}\cdot MEKi\right)}^{k}}{\tau +\;{\left(w_{akt}\cdot AKTi+\;w_{erk}\cdot MEKi\right)}^{k}}\right)$$

*Pathway Bias* was then defined as the normalize differential between the parameters *w*_*akt*_ and *w*_*erk*_:

$$Bias\;=\;\frac{\left(w_{akt}-\;w_{erk}\right)}{\left(w_{akt}+w_{erk}\right)}$$

Based on our finding that PI3K/AKT dependence correlated with the cell death rate (*δ*_*MAX*_), and MAPK-dependence with proliferation (*μ*_*MAX*_), we created a tenth model (M10) which separates the regulatory terms accordingly:

$$\frac{dX}{dt}=\mu_{MAX}\left(1-\frac{{MEKi}^{k\_erk}}{\tau_{erk}+\;{MEKi}^{k\_erk}}\right)-\;\delta_{MAX}\left(\frac{{AKTi}^{k\_akt}}{\tau_{akt}+\;{AKTi}^{k\_akt}}\right)$$

The raw cell growth data, model parameters associated with each of the ten models (M1-M10), goodness-of-fit metrics (MSE and AIC) and simulations are provided in **S1 Data**. Parameter estimates across alternate models are quite consistent, indicating our results are robust regardless of the model chosen.

### Logistic regression models of Pathway Bias

The Pathway Bias measurement for each cell was first discretized into MAPK vs. PI3K-dependence (*Bias* = -1 vs. +1), a reasonable simplification given the observed bimodal distribution of this metric. The probability of MAPK-dependence (*P*_*MAPK*_) vs. PI3K-dependence (*P*_*PI3K*_ = 1 –*P*_*MAPK*_) was then modelled as a function of input features using a logistic regression equation:

$$\text{ln}\;\left(\frac{P_{MAPK}}{P_{PI3K}}\right)=\;\beta_{0}+\sum_{i=1}^{N}\beta_{i}\cdot X_{i}$$

Where *N* = number of features (*X*_*i*_) and *β*_*i*_ = regression coefficients. The *β*_*i*_ parameters were estimated by maximum likelihood estimation, and predictive power of the model assessed using leave-one-out cross validation (LOOCV) procedure. Model-predicted Bias was then back-calculated using the probabilities as:

$$\mathrm{Predicted Bias}=-1\cdot P_{\mathrm{MAPK}}+1\cdot P_{\mathrm{PI}3K}$$

Statistical significance of model predictions was assessed by computing LOOCV accuracy, Pearson correlations, and mean squared error (MSE) from 10,000 randomized permutations of the cellular properties: Bias mapping.

### TCGA analysis

RNAseq was downloaded from the GDAC Firehose portal in June, 2014 (http://gdac.broadinstitute.org/). HER2+/- classifications were based on *ERBB2* expression. Using BRCA, LUAD, and OV samples as controls, setting *ERBB2* RNAseq count thresholds at 14,000 resulted in HER2+ frequencies consistent with known ERBB2-amplification frequencies of 13%, 2.5%, and 1.5%. This threshold was then applied across all indications, though results were insensitive to the specific value chosen.

### Cell line data sources and analysis

mRNA expression data was downloaded from CCLE (www.broadinstitute.org/ccle/home) and gene knockdown sensitivity from Project Achilles (www.broadinstitute.org/achilles). Signaling networks were defined in NCI-PID (http://pid.nci.nih.gov/index.shtml) and accessed via the Pathway Commons portal (www.pathwaycommons.org), and visualized using Cytoscape (www.cytoscape.org).

All analysis and simulations were carried out in MATLAB R2013b.

## Supporting Information

S1 Fig Cell surface response plots of cell population doubling (PD) 96-hour following treatment with AKT (MK2206) and MEK (GSK1120212) inhibitor matrices. Cell surface responses from AU565, BT474-M3, CALU3, HCC202, HCC1419, HCC1954, JIMT1 and MDAMB175VII are depicted in individual boxes. Top panels show data from FBS-supplemented media, and bottom panels with 2nM heregulin ligand (HRG) addition. Left plots are the raw data, middle column the OR-gate model (“M4”) used in our analysis, and right plots the optimal logic model, as determined by MSE minimization.
(PPTX)

S2 Fig Cell surface response plots of cell population doubling (PD) 96-hour following treatment with AKT (MK2206) and MEK (GSK1120212) inhibitor matrices. Cell surface responses from MDAMB361, MDAMB453, NCI-N87, NCI-H2170, OE19, OE33, SKBR3 and SKOV3 are depicted in individual boxes. Top panels show data from FBS-supplemented media, and bottom panels with 2nM heregulin ligand (HRG) addition. Left plots are the raw data, middle column the OR-gate model (“M4”) used in our analysis, and right plots the optimal logic model, as determined by MSE minimization.
(PPTX)

S3 Fig Cell surface response plots of cell population doubling (PD) 96-hour following treatment with AKT (MK2206) and MEK (GSK1120212) inhibitor matrices. Cell surface responses from ZR75-1 and ZR75-30 are depicted in individual boxes. Top panels show data from FBS-supplemented media, and bottom panels with 2nM heregulin ligand (HRG) addition. Left plots are the raw data, middle column the OR-gate model (“M4”) used in our analysis, and right plots the optimal logic model, as determined by MSE minimization.
(PPTX)

S4 Fig Quantitative logic-based models of cell growth regulation by ERK and AKT signals. **(A)** Generic logic model; combinations of OR, AND, or null (K) gates can be combined to describe signal-response relationships. **(B)** Quantitative comparison of 9 alternate model forms across all cell lines, based on Akaike Information Criterion (AIC) minimization. **(C)** Weighted sum of squared residual (WSSR; the objective function) for each cell line, shown for the Best, worst, and M4 (OR gate) functions. **(D)** Pearson correlation coefficients between model vs. raw data for all 18 cell lines using the “M4” OR-gate function.
(PPTX)

S5 Fig Select model parameter estimates and precision for each cell line +/- heregulin (HRG) stimulation. AKT and ERK weights for each cell line +/- HRG are shown in (A, B). (C) Median coefficient of variation (CV) +/- 1 standard deviation across the cell line panel for each of the four model parameters, the relations (Pathway Bais, and u_max_/d_max_) as well as the fitting metrics AIC and MSE.
(PPTX)

S6 Fig Basal protein expression (column normalized) for the 18 cell lines +/- heregulin (HRG). Data is represented using a hierarchical clustered heatmap, and PI3K, MAPK, and SWITCH classification scheme color-coded for each cell.
(PPTX)

S7 Fig Heregulin-induced changes in protein expression across cell lines. Data is represented as z-scores (heregulin-stimulated vs. FBS), and cell lines classified into PI3K, MAPK, and SWITCH categories
(PPTX)

S8 Fig Multivariate logistic model-based classification of Pathway Bias. Comparison of logistic model accuracy built using alternate sets of input features. Predictions from alternate models were evaluated via Pearson correlation coefficients, Mean Squared Error (MSE), and accuracy as compared to randomized data.
(PPTX)

S9 Fig Rank correlation coefficients between expressions of protein and mRNA (A) and mRNA-mRNA (B). mRNA expression is taken from the CCLE database (RMA values), and protein expression from two sources; our 18-cell line Luminex profile (18 HER2+) and an internal ELISA-based profile (MBASE) segregated into HER2+ and all cells.
(PPTX)

S10 Fig Expression of ERBB3, EGFR, and CDKN1B across multiple HER2+ cancer indications represented in TCGA. (A) Raw RNAseq counts are displayed for all indications, and HER2+ defined as samples with greater than 15,000 counts. (B) Differential expression for each gene compared to breast cancers, separated by HER2+ and HER2- populations.
(PPTX)

S11 Fig Spearman’s Rank Correlation coefficients between sensitivities to select pathway inhibitors across all cell lines in the GDSC database. Asterisks indicate drugs identified as differentially sensitive between predicted PI3K (blue) and MAPK (red) cell subsets.
(PPTX)

S1 Table Summary of logic-based cell growth models evaluated. (DOCX)

S2 Table “M4” Model Parameters and associated error. (DOCX)

S3 Table A) Capture antibodies used in Luminex assays. B) Detection antibodies used in Luminex assays.
(DOCX)

S4 Table Logistic model parameters, wherein median values and standard deviations are taken from the 36 LOOCV estimates. (DOCX)

S5 Table Raw Protein Signals, and Logistic Model Predictions of Pathway Bias. (DOCX)

S6 Table Evaluation of EGFR, ERBB3, pAKT, and pERK protein expression as predictive biomarkers for HDACi, PI3K/AKT/MTORi, and MEKi sensitivity in HER2+ cell represented in the GDSC Database. (DOCX)

S1 Data Excel spreadsheet containing mean cell growth response data, Luminex protein signals, logic model parameter estimates and simulations. (XLSX)

S2 Data Statistics associated with *EGFR*, *ERBB3*, and *CDKN1B* mRNA expression, and *PIK3CA* mutation-based stratification of shRNA knockdown data from Project Achilles. (XLSX)
