# Supplementary figures and images for: HER2+ Cancer Cell Dependence on PI3K vs. MAPK Signaling Axes Is Determined by Expression of EGFR, ERBB3 and CDKN1B
Source: PLoS Comput Biol. 2016 Apr 1;12(4):e1004827. doi: 10.1371/journal.pcbi.1004827 (PMC4818107; doi:10.1371/journal.pcbi.1004827)

## Slide 1
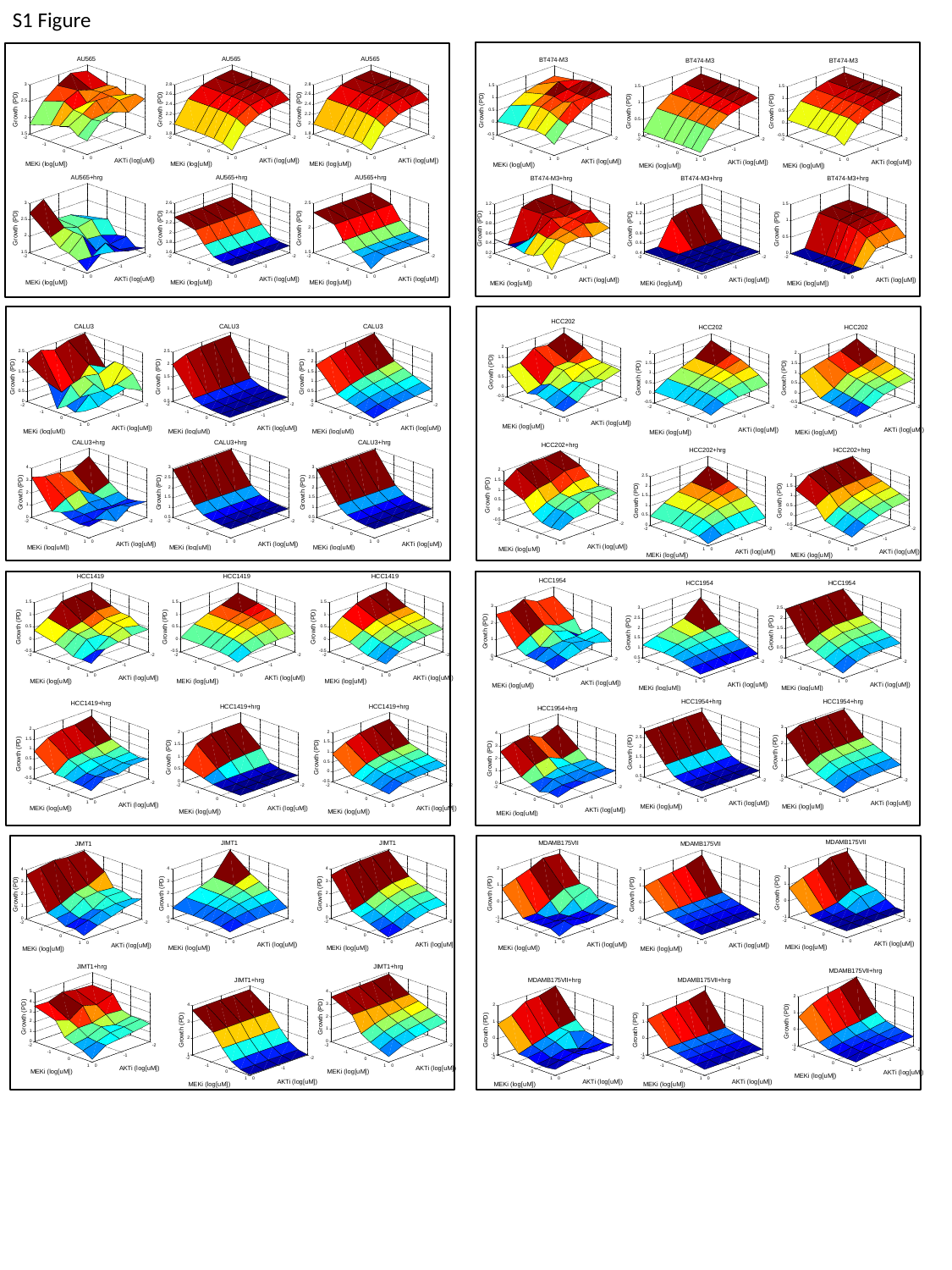

S1 Figure

Supplement: S1 Fig — Cell surface responses from AU565, BT474-M3, CALU3, HCC202, HCC1419, HCC1954, JIMT1 and MDAMB175VII are depicted in individual boxes. Top panels show data from FBS-supplemented media, and bottom panels with 2nM heregulin ligand (HRG) addition. Left plots are the raw data, middle column the OR-gate model (“M4”) used in our analysis, and right plots the optimal logic model, as determined by MSE minimization. (PPTX) [file pcbi.1004827.s001.pptx]

## Slide 1
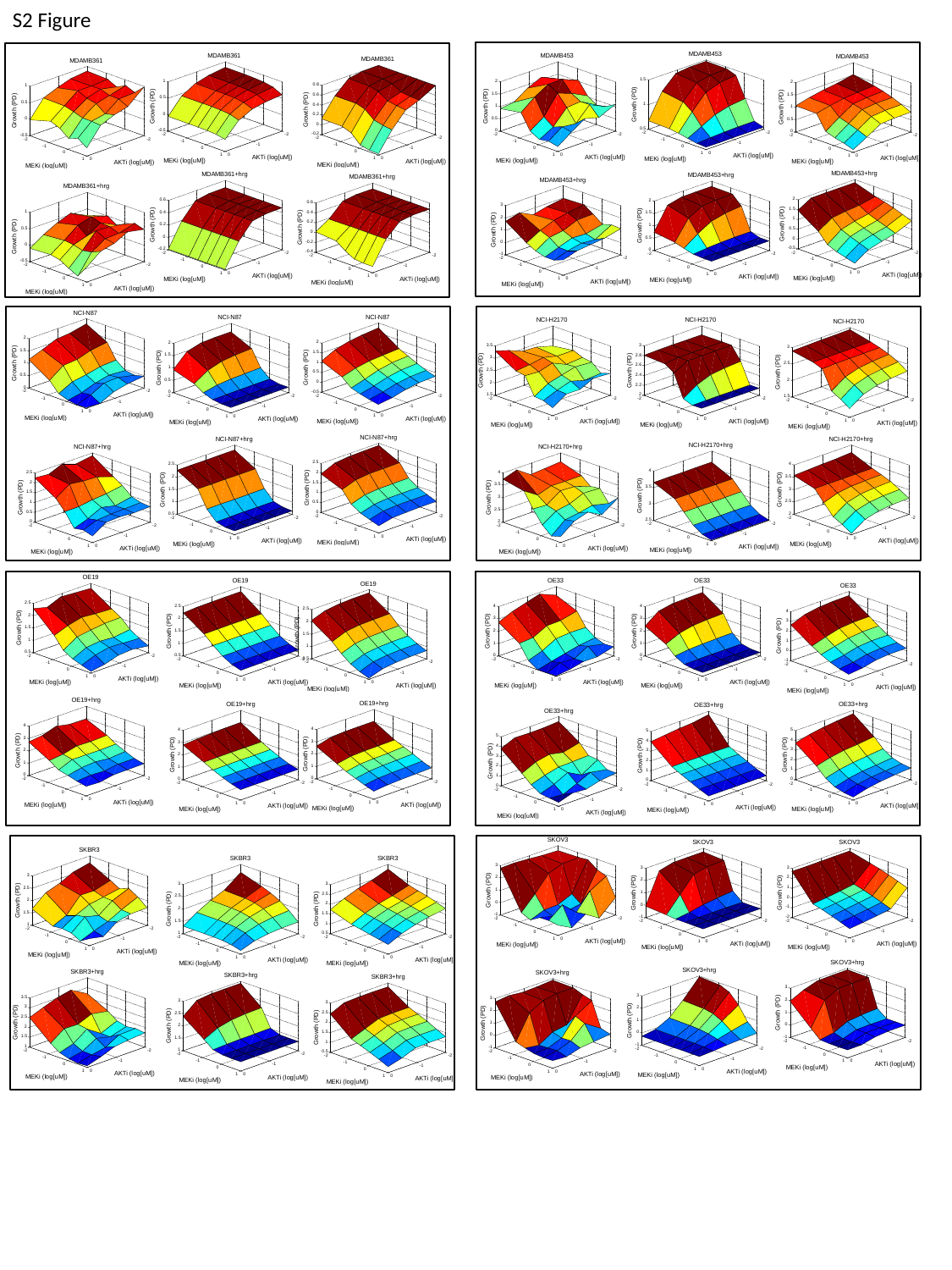

S2 Figure

Supplement: S2 Fig — Cell surface responses from MDAMB361, MDAMB453, NCI-N87, NCI-H2170, OE19, OE33, SKBR3 and SKOV3 are depicted in individual boxes. Top panels show data from FBS-supplemented media, and bottom panels with 2nM heregulin ligand (HRG) addition. Left plots are the raw data, middle column the OR-gate model (“M4”) used in our analysis, and right plots the optimal logic model, as determined by MSE minimization. (PPTX) [file pcbi.1004827.s002.pptx]

## Slide 1
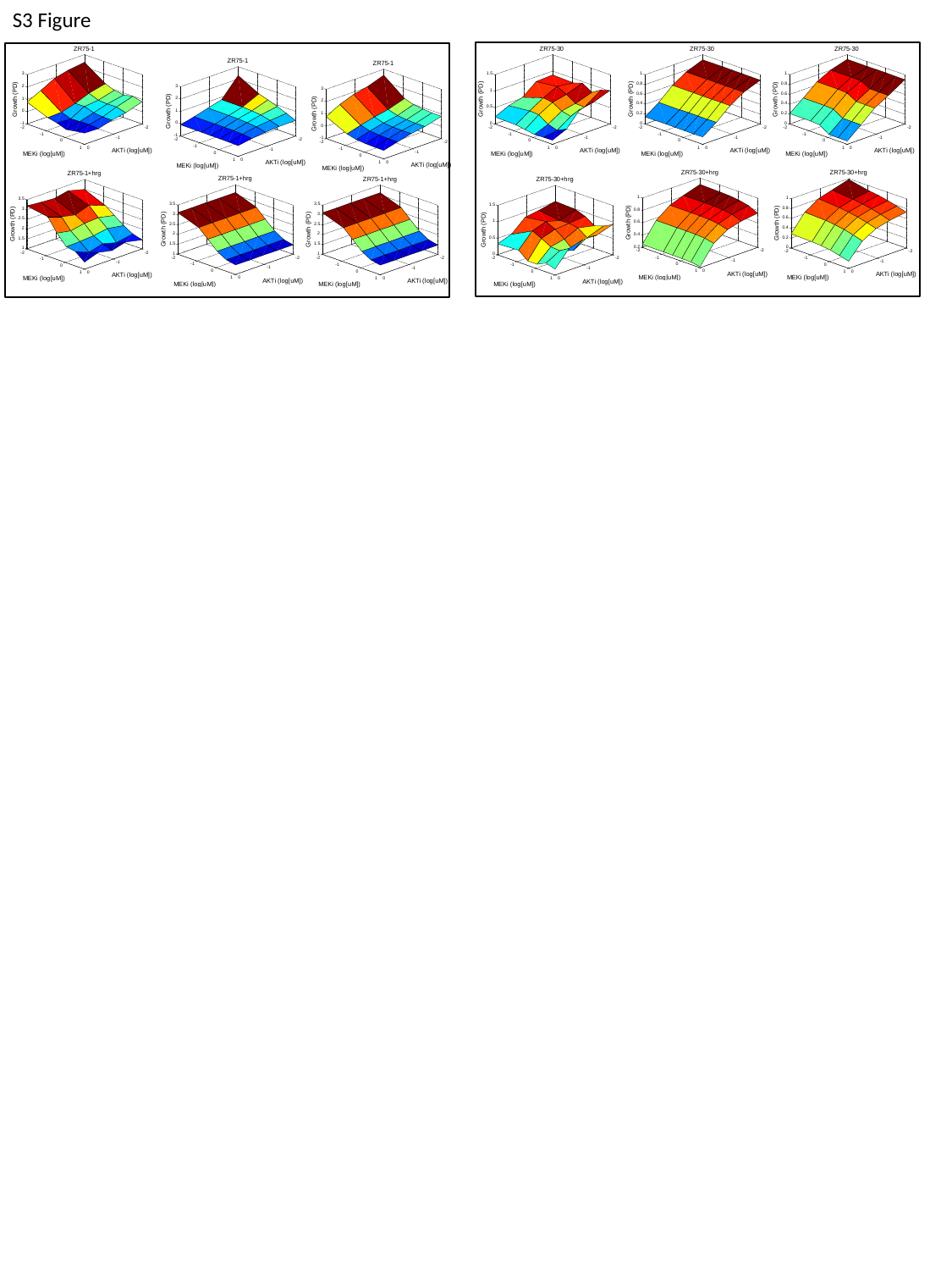

S3 Figure

Supplement: S3 Fig — Cell surface responses from ZR75-1 and ZR75-30 are depicted in individual boxes. Top panels show data from FBS-supplemented media, and bottom panels with 2nM heregulin ligand (HRG) addition. Left plots are the raw data, middle column the OR-gate model (“M4”) used in our analysis, and right plots the optimal logic model, as determined by MSE minimization. (PPTX) [file pcbi.1004827.s003.pptx]

## Slide 1
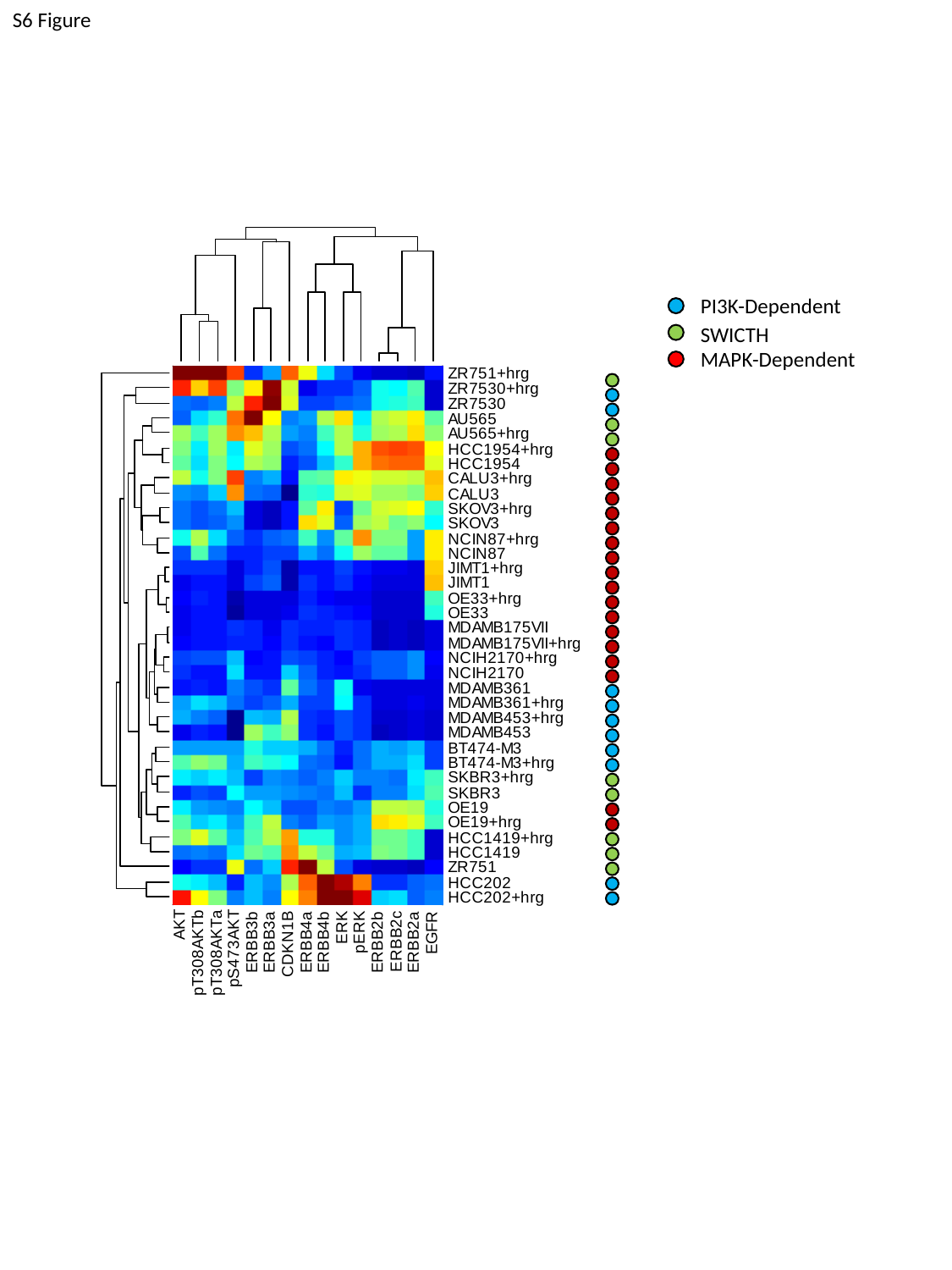

S6 Figure
PI3K-Dependent
SWICTH
MAPK-Dependent

Supplement: S6 Fig — Data is represented using a hierarchical clustered heatmap, and PI3K, MAPK, and SWITCH classification scheme color-coded for each cell. (PPTX) [file pcbi.1004827.s006.pptx]

## Slide 1
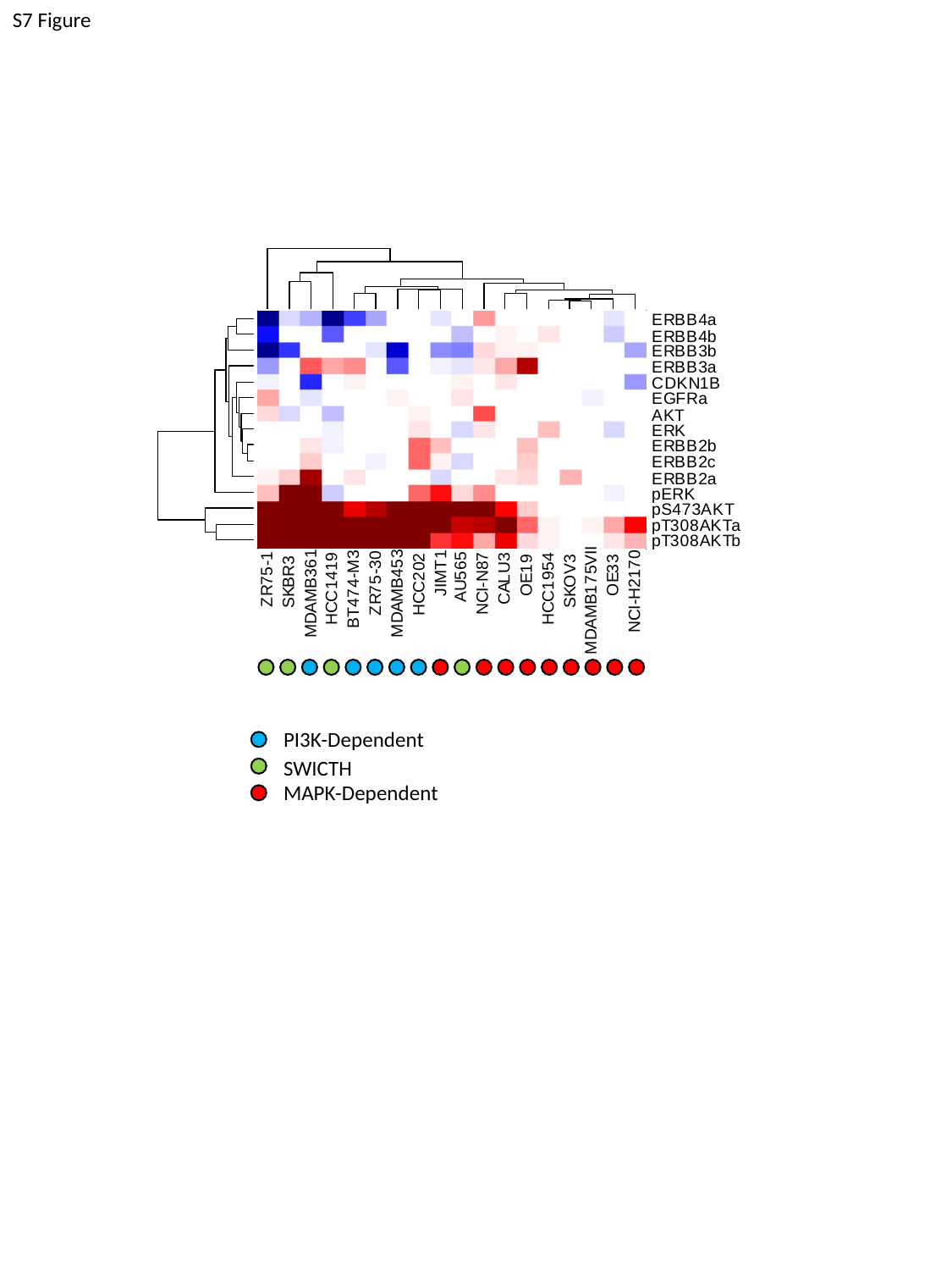

S7 Figure
PI3K-Dependent
SWICTH
MAPK-Dependent

Supplement: S7 Fig — Data is represented as z-scores (heregulin-stimulated vs. FBS), and cell lines classified into PI3K, MAPK, and SWITCH categories (PPTX) [file pcbi.1004827.s007.pptx]

## Slide 1
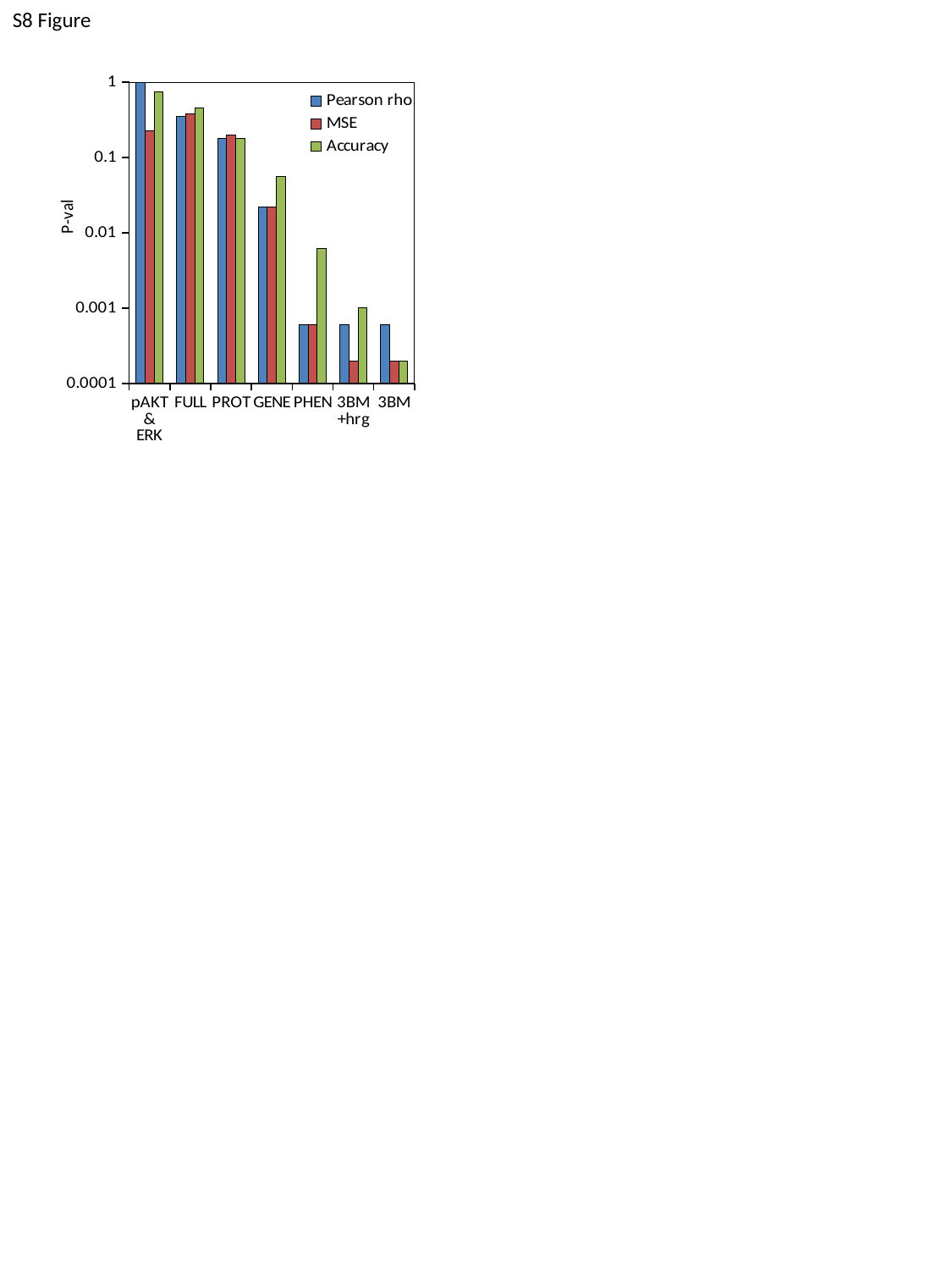

S8 Figure
### Chart
| Category | | | |
|---|---|---|---|
| pAKT & ERK | 0.9776 | 0.22719999999999999 | 0.7486 |
| FULL | 0.35 | 0.3824 | 0.45299999999999996 |
| PROT | 0.17980000000000002 | 0.1998 | 0.17859999999999998 |
| GENE | 0.0222 | 0.0222 | 0.0553999999999999 |
| PHEN | 0.000600000000000023 | 0.000600000000000023 | 0.0061999999999999 |
| 3BM+hrg | 0.000600000000000023 | 0.0002 | 0.000999999999999943 |
| 3BM | 0.000600000000000023 | 0.0002 | 0.00019999999999996 |

Supplement: S8 Fig — Comparison of logistic model accuracy built using alternate sets of input features. Predictions from alternate models were evaluated via Pearson correlation coefficients, Mean Squared Error (MSE), and accuracy as compared to randomized data. (PPTX) [file pcbi.1004827.s008.pptx]

## Slide 1
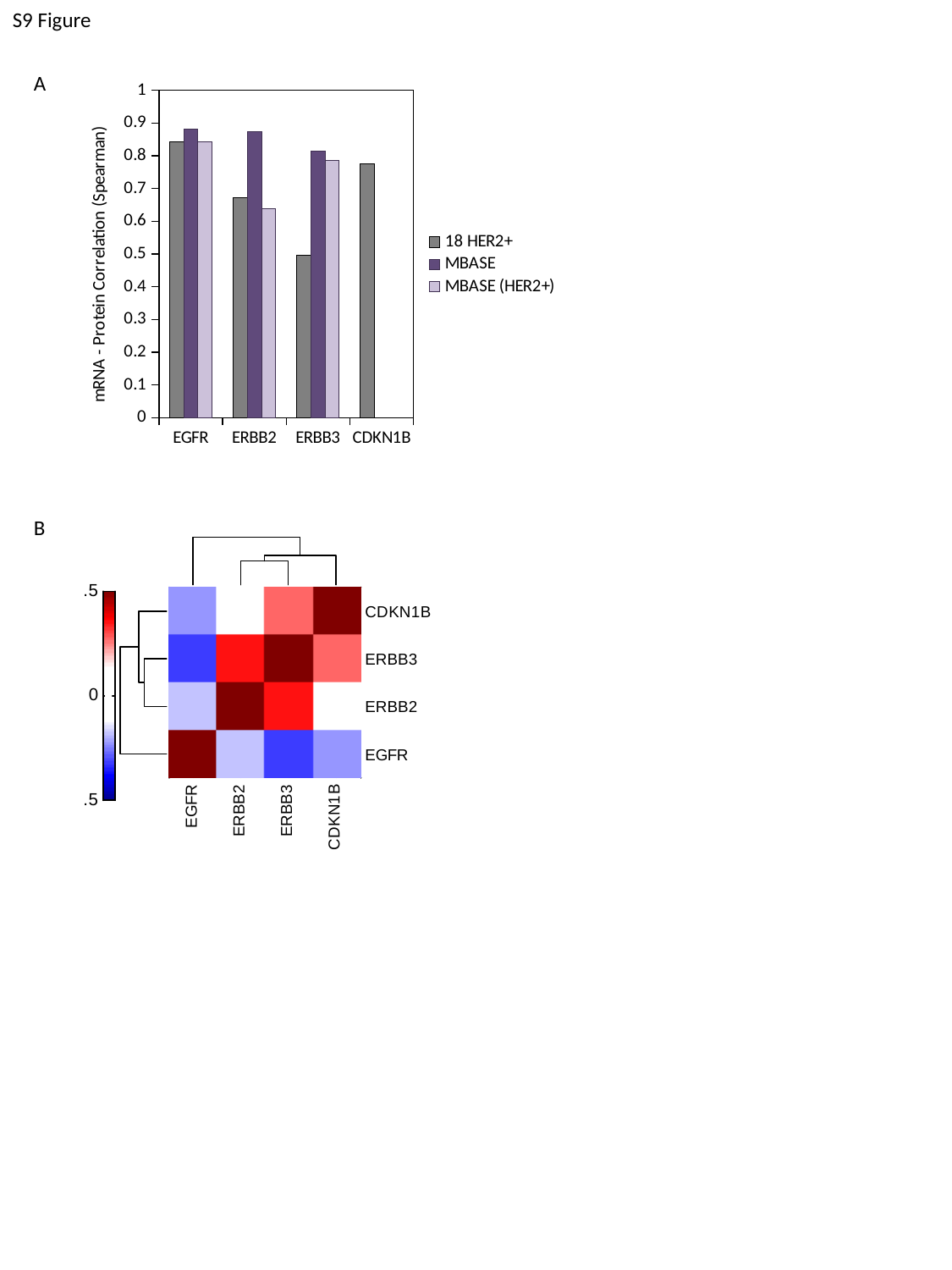

S9 Figure
A
### Chart
| Category | | | |
|---|---|---|---|
| EGFR | 0.843654358449439 | 0.881665801219379 | 0.84297520661157 |
| ERBB2 | 0.6715686274509807 | 0.874036762934996 | 0.637362637362637 |
| ERBB3 | 0.496627929029103 | 0.81433176250822 | 0.785714285714286 |
| CDKN1B | 0.774509803921569 | None | None |
B

Supplement: S9 Fig — mRNA expression is taken from the CCLE database (RMA values), and protein expression from two sources; our 18-cell line Luminex profile (18 HER2+) and an internal ELISA-based profile (MBASE) segregated into HER2+ and all cells. (PPTX) [file pcbi.1004827.s009.pptx]

## Slide 1
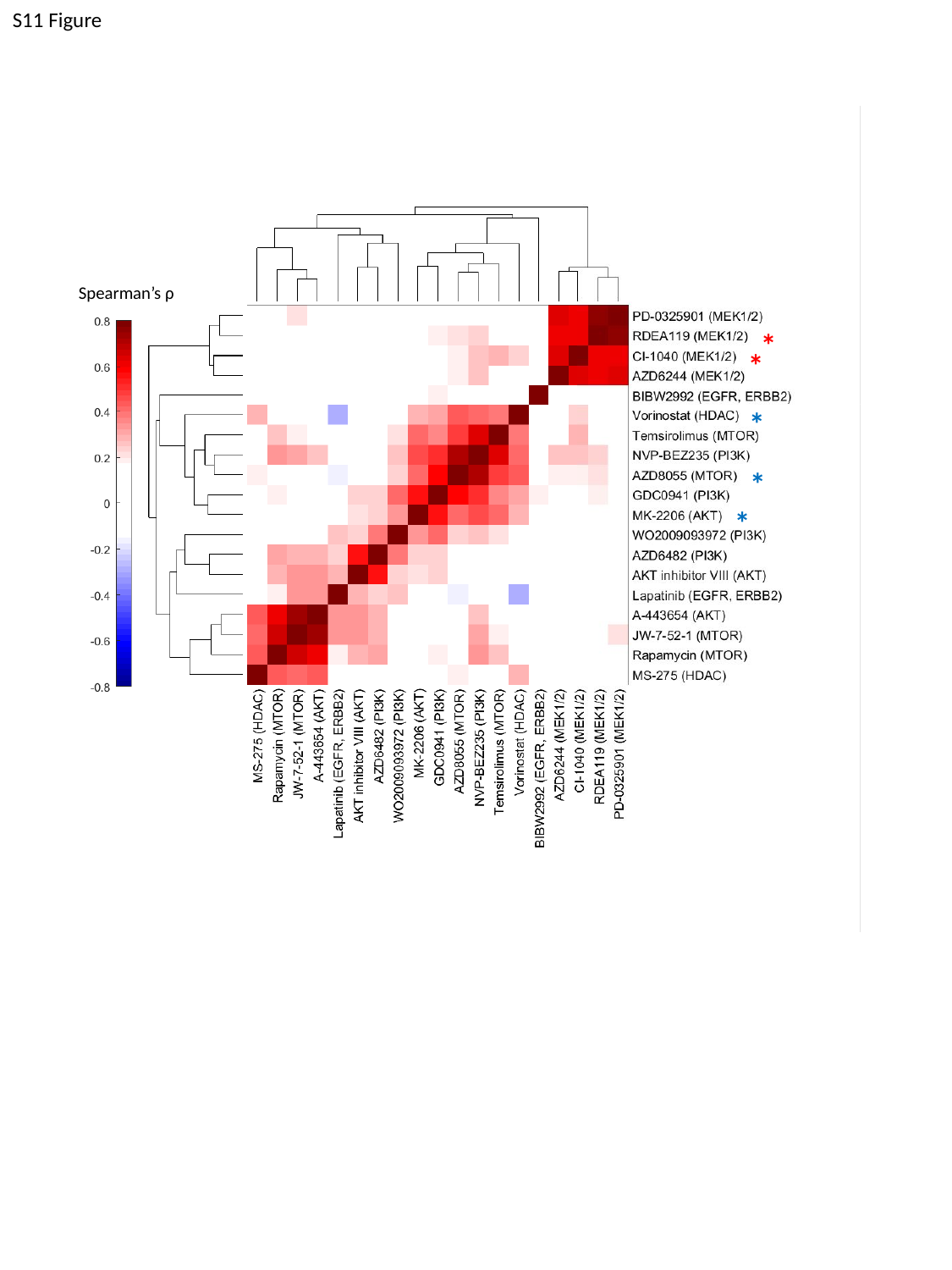

S11 Figure
*
*
*
*
*
Spearman’s ρ

Supplement: S11 Fig — Asterisks indicate drugs identified as differentially sensitive between predicted PI3K (blue) and MAPK (red) cell subsets. (PPTX) [file pcbi.1004827.s011.pptx]
