# Supplementary material for: HER2+ Cancer Cell Dependence on PI3K vs. MAPK Signaling Axes Is Determined by Expression of EGFR, ERBB3 and CDKN1B
Source: PLoS Comput Biol. 2016 Apr 1;12(4):e1004827. doi: 10.1371/journal.pcbi.1004827 (PMC4818107; doi:10.1371/journal.pcbi.1004827)
Supplement: S4 Fig — (A) Generic logic model; combinations of OR, AND, or null (K) gates can be combined to describe signal-response relationships. (B) Quantitative comparison of 9 alternate model forms across all cell lines, based on Akaike Information Criterion (AIC) minimization. (C) Weighted sum of squared residual (WSSR; the objective function) for each cell line, shown for the Best, worst, and M4 (OR gate) functions. (D) Pearson correlation coefficients between model vs. raw data for all 18 cell lines using the “M4” OR-gate function. (PPTX) [file pcbi.1004827.s004.pptx]

## Slide 1
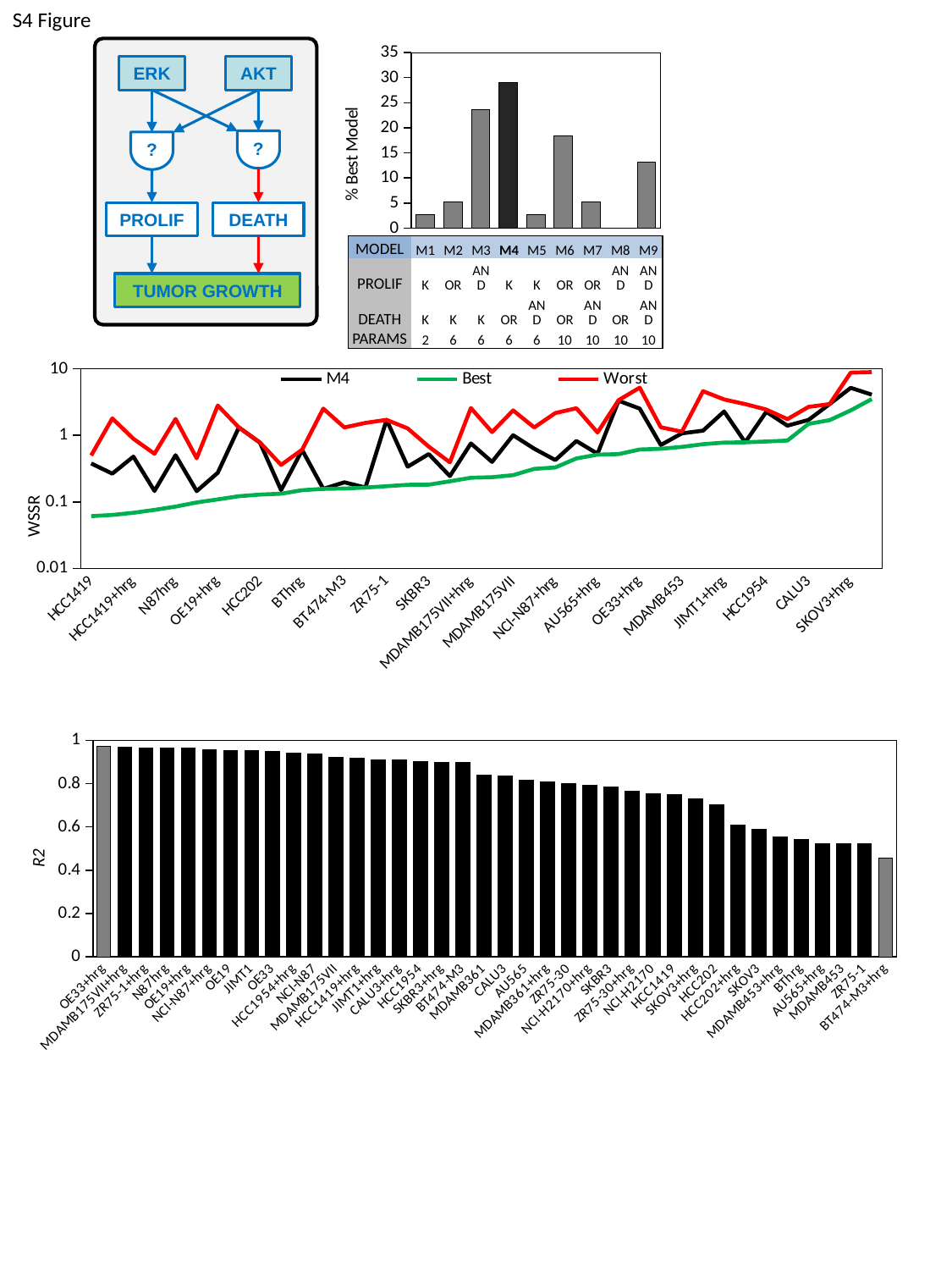

S4 Figure
### Chart
| Category | |
|---|---|
| M1: K-K | 2.631578947368421 |
| M2: OR-K | 5.263157894736842 |
| M3: AND-K | 23.684210526315788 |
| M4: K-OR | 28.947368421052634 |
| M5: K-AND | 2.631578947368421 |
| M6: OR-OR | 18.421052631578945 |
| M7: OR-AND | 5.263157894736842 |
| M8: AND-OR | 0.0 |
| M9: AND-AND | 13.157894736842104 |
ERK
AKT
?
?
PROLIF
DEATH
TUMOR GROWTH
| MODEL | M1 | M2 | M3 | M4 | M5 | M6 | M7 | M8 | M9 |
| --- | --- | --- | --- | --- | --- | --- | --- | --- | --- |
| PROLIF | K | OR | AND | K | K | OR | OR | AND | AND |
| DEATH | K | K | K | OR | AND | OR | AND | OR | AND |
| PARAMS | 2 | 6 | 6 | 6 | 6 | 10 | 10 | 10 | 10 |
### Chart
| Category | | | |
|---|---|---|---|
| HCC1419 | 0.377994843539835 | 0.0611319336997699 | 0.498941273143782 |
| OE19 | 0.265560412824079 | 0.0637158912484095 | 1.79729173076177 |
| HCC1419+hrg | 0.47839534013816 | 0.0685879145937654 | 0.888574509155749 |
| MDAMB361 | 0.146106212534582 | 0.0758045473066599 | 0.529130547365475 |
| N87hrg | 0.503079884083584 | 0.0850020434434114 | 1.76338569129465 |
| ZR75-30 | 0.145444410292136 | 0.0980893240003049 | 0.452526265619233 |
| OE19+hrg | 0.274345302154357 | 0.109072441726103 | 2.80521159431537 |
| HCC202+hrg | 1.31434969471476 | 0.12189212147598 | 1.31434969471476 |
| HCC202 | 0.775650133040136 | 0.128854032428162 | 0.781508100703364 |
| MDAMB361+hrg | 0.150187336939341 | 0.132927053113019 | 0.35993991032346 |
| BThrg | 0.602176920883486 | 0.149881709617316 | 0.609842301986551 |
| ZR75-1+hrg | 0.157228987108275 | 0.157228987108275 | 2.52352169022389 |
| BT474-M3 | 0.196654713727113 | 0.158844790401547 | 1.31464765198777 |
| AU565 | 0.164597162604867 | 0.164436396694822 | 1.53836198356053 |
| ZR75-1 | 1.70804521772947 | 0.172233830908339 | 1.70804531219511 |
| NCI-N87 | 0.338173130123933 | 0.180572211558111 | 1.27167568741194 |
| SKBR3 | 0.523402980928086 | 0.181758130831092 | 0.676032730291606 |
| ZR75-30+hrg | 0.244005626881766 | 0.204018131649127 | 0.393325291411445 |
| MDAMB175VII+hrg | 0.755380655763627 | 0.23050259538453 | 2.57062623074264 |
| NCI-H2170 | 0.398738105991021 | 0.235115545017018 | 1.11839161946494 |
| MDAMB175VII | 1.00505640858655 | 0.252144029883763 | 2.36916683964439 |
| NCI-H2170+hrg | 0.630170522330968 | 0.313311161339641 | 1.31373878294275 |
| NCI-N87+hrg | 0.425419597861455 | 0.329311274717452 | 2.16092590039848 |
| HCC1954+hrg | 0.822548332218924 | 0.449841554336617 | 2.55455474326845 |
| AU565+hrg | 0.529777443661162 | 0.512851388515774 | 1.10398854973134 |
| JIMT1 | 3.31025152853802 | 0.522486266229967 | 3.37523114322491 |
| OE33+hrg | 2.52216443346652 | 0.612459580791123 | 5.20591603955394 |
| SKBR3+hrg | 0.716305749041526 | 0.62776346622567 | 1.32032235327076 |
| MDAMB453 | 1.07173917396286 | 0.670600341319097 | 1.13365744397036 |
| OE33 | 1.17591005841183 | 0.736053529287047 | 4.60136242650781 |
| JIMT1+hrg | 2.28670945442852 | 0.777413709049009 | 3.45878173384412 |
| CALU3+hrg | 0.785886168196989 | 0.785886168196989 | 2.94698958729485 |
| HCC1954 | 2.25942838730738 | 0.80789045885163 | 2.44061422869952 |
| MDAMB453+hrg | 1.39882794482281 | 0.835125112583829 | 1.75026021399079 |
| CALU3 | 1.70605112036938 | 1.48470231466412 | 2.67490337863891 |
| BT474-M3+hrg | 2.94482114392935 | 1.69180176717277 | 2.94482114392935 |
| SKOV3+hrg | 5.16575681673546 | 2.37037715791786 | 8.75054622418118 |
| SKOV3 | 4.08287614597989 | 3.50928038706107 | 8.91617952619843 |
### Chart
| Category | |
|---|---|
| OE33+hrg | 0.9735032248549856 |
| MDAMB175VII+hrg | 0.9680930315867072 |
| ZR75-1+hrg | 0.9657339658589335 |
| N87hrg | 0.964703175201558 |
| OE19+hrg | 0.9632012534580466 |
| NCI-N87+hrg | 0.9560577697808664 |
| OE19 | 0.9523002780290715 |
| JIMT1 | 0.9514389319690685 |
| OE33 | 0.9486193376930744 |
| HCC1954+hrg | 0.9402135035995483 |
| NCI-N87 | 0.9382728981300108 |
| MDAMB175VII | 0.9194185467628323 |
| HCC1419+hrg | 0.9162862693408439 |
| JIMT1+hrg | 0.9088004141269933 |
| CALU3+hrg | 0.9076773063921982 |
| HCC1954 | 0.8999265451136053 |
| SKBR3+hrg | 0.8975424053340175 |
| BT474-M3 | 0.8973643265283915 |
| MDAMB361 | 0.839226638597159 |
| CALU3 | 0.8332743471742741 |
| AU565 | 0.8153999065436437 |
| MDAMB361+hrg | 0.806062672019797 |
| ZR75-30 | 0.7983961572613383 |
| NCI-H2170+hrg | 0.791869050451376 |
| SKBR3 | 0.7849092037593947 |
| ZR75-30+hrg | 0.7658951963025886 |
| NCI-H2170 | 0.7530094984726267 |
| HCC1419 | 0.7484226107646929 |
| SKOV3+hrg | 0.7313485267810721 |
| HCC202 | 0.7036787209512367 |
| HCC202+hrg | 0.6097450145607497 |
| SKOV3 | 0.5884390318455405 |
| MDAMB453+hrg | 0.5548815010585468 |
| BThrg | 0.5405803014194354 |
| AU565+hrg | 0.5231122431556785 |
| MDAMB453 | 0.5218646319775647 |
| ZR75-1 | 0.5206500752200088 |
| BT474-M3+hrg | 0.4567257273259304 |
