# Supplementary material for: HER2+ Cancer Cell Dependence on PI3K vs. MAPK Signaling Axes Is Determined by Expression of EGFR, ERBB3 and CDKN1B
Source: PLoS Comput Biol. 2016 Apr 1;12(4):e1004827. doi: 10.1371/journal.pcbi.1004827 (PMC4818107; doi:10.1371/journal.pcbi.1004827)
Supplement: S5 Fig — AKT and ERK weights for each cell line +/- HRG are shown in (A, B). (C) Median coefficient of variation (CV) +/- 1 standard deviation across the cell line panel for each of the four model parameters, the relations (Pathway Bais, and umax/dmax) as well as the fitting metrics AIC and MSE. (PPTX) [file pcbi.1004827.s005.pptx]

## Slide 1
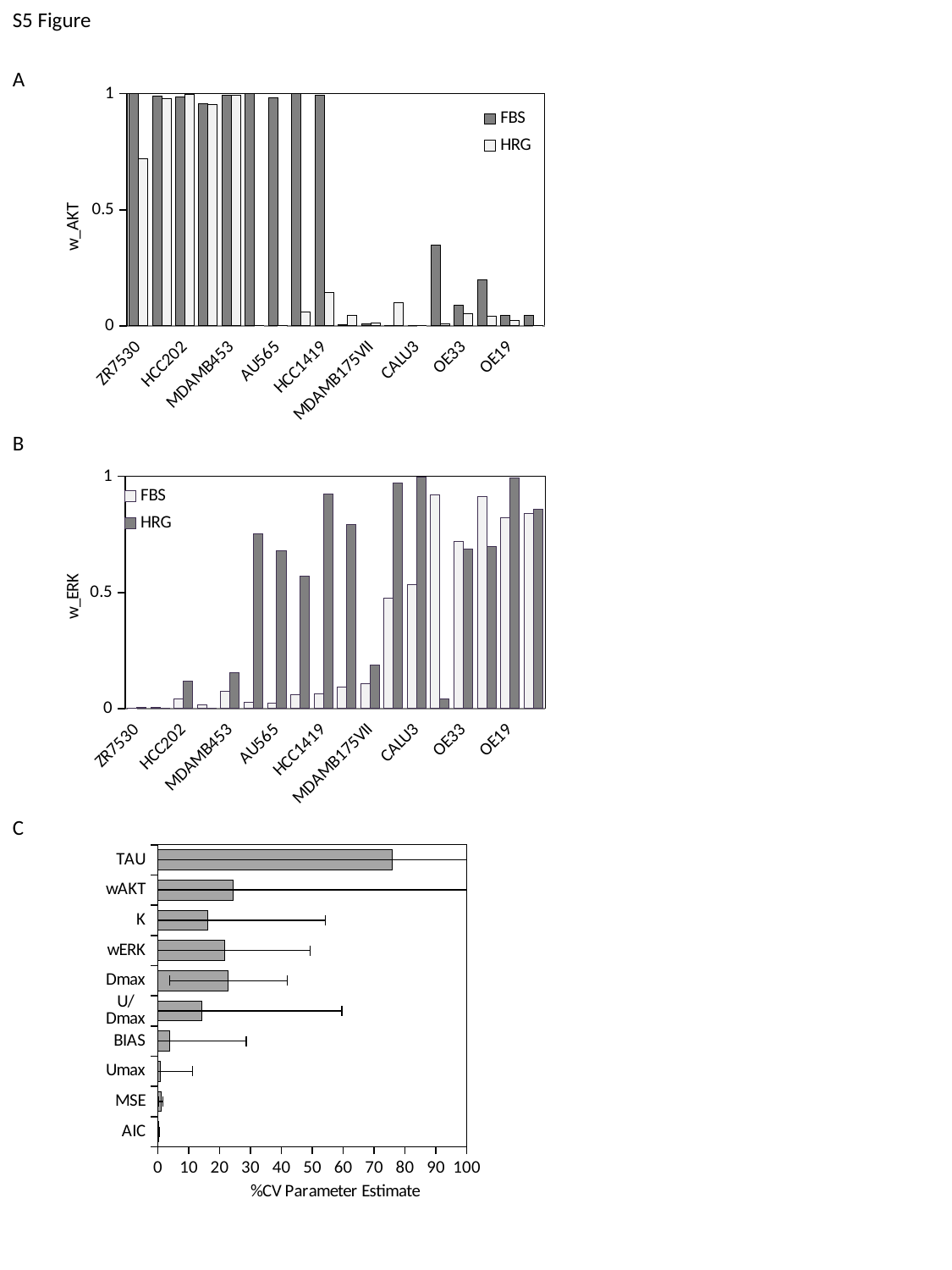

S5 Figure
A
### Chart
| Category | | |
|---|---|---|
| ZR7530 | 1.0 | 0.7197647114722 |
| BT474-M3 | 0.991155765887478 | 0.976749441151959 |
| HCC202 | 0.984199623108249 | 0.995696185776378 |
| MDAMB361 | 0.957986824613491 | 0.954680564365119 |
| MDAMB453 | 0.994229026749459 | 0.994595237708044 |
| ZR751 | 1.0 | 0.000968559656283212 |
| AU565 | 0.982509417432452 | 0.0001 |
| SKBR3 | 0.999841260161366 | 0.0578834240388526 |
| HCC1419 | 0.993706153157586 | 0.142524116995478 |
| HCC1954 | 0.0047604436575773 | 0.043197229900868 |
| MDAMB175VII | 0.00896776285763077 | 0.0119915420125852 |
| JIMT1 | 0.000221604177824378 | 0.100816360666265 |
| CALU3 | 0.00238059113308497 | 0.000100014591697573 |
| SKOV3** | 0.345914636597001 | 0.00992529526555754 |
| OE33 | 0.087639738007352 | 0.0538008643368362 |
| NCIN87 | 0.198933218651553 | 0.0412871711718893 |
| OE19 | 0.0455169936452811 | 0.0228047024386557 |
| NCIH2170 | 0.0429047597843143 | 0.0001 |B
### Chart
| Category | | |
|---|---|---|
| ZR7530 | 0.000102676471732279 | 0.00608452345492636 |
| BT474-M3 | 0.00476916692180994 | 0.00216399718968307 |
| HCC202 | 0.0395989366615811 | 0.116887177630493 |
| MDAMB361 | 0.014644401286021 | 0.000125103739424223 |
| MDAMB453 | 0.0733080476434262 | 0.152668427183369 |
| ZR751 | 0.0249601842162423 | 0.753377902916214 |
| AU565 | 0.0229038506834394 | 0.681019306753105 |
| SKBR3 | 0.0602329773640598 | 0.569518537586823 |
| HCC1419 | 0.0635376954817781 | 0.923307517009382 |
| HCC1954 | 0.0933411661313135 | 0.792308017463304 |
| MDAMB175VII | 0.107055312037477 | 0.187856487985352 |
| JIMT1 | 0.47543724286995 | 0.970696328473544 |
| CALU3 | 0.534618941314595 | 0.99624574380529 |
| SKOV3** | 0.918843621225337 | 0.0399771399939502 |
| OE33 | 0.720342916831214 | 0.685694281778743 |
| NCIN87 | 0.914147709300645 | 0.696333210051015 |
| OE19 | 0.820922626286188 | 0.991691271083994 |
| NCIH2170 | 0.840306813879858 | 0.856393456913413 |C
### Chart
| Category | |
|---|---|
| AIC | 0.18477192390221742 |
| MSE | 0.9338686579858837 |
| Umax | 0.7904811488416794 |
| BIAS | 3.7403885023991026 |
| U/Dmax | 14.240505196772201 |
| Dmax | 22.76585394273956 |
| wERK | 21.704653371110393 |
| K | 16.079691559828788 |
| wAKT | 24.406564068282965 |
| TAU | 75.7658747064506 |
