# Supplementary material for: HER2+ Cancer Cell Dependence on PI3K vs. MAPK Signaling Axes Is Determined by Expression of EGFR, ERBB3 and CDKN1B
Source: PLoS Comput Biol. 2016 Apr 1;12(4):e1004827. doi: 10.1371/journal.pcbi.1004827 (PMC4818107; doi:10.1371/journal.pcbi.1004827)
Supplement: S10 Fig — (A) Raw RNAseq counts are displayed for all indications, and HER2+ defined as samples with greater than 15,000 counts. (B) Differential expression for each gene compared to breast cancers, separated by HER2+ and HER2- populations. (PPTX) [file pcbi.1004827.s010.pptx]

## Slide 1
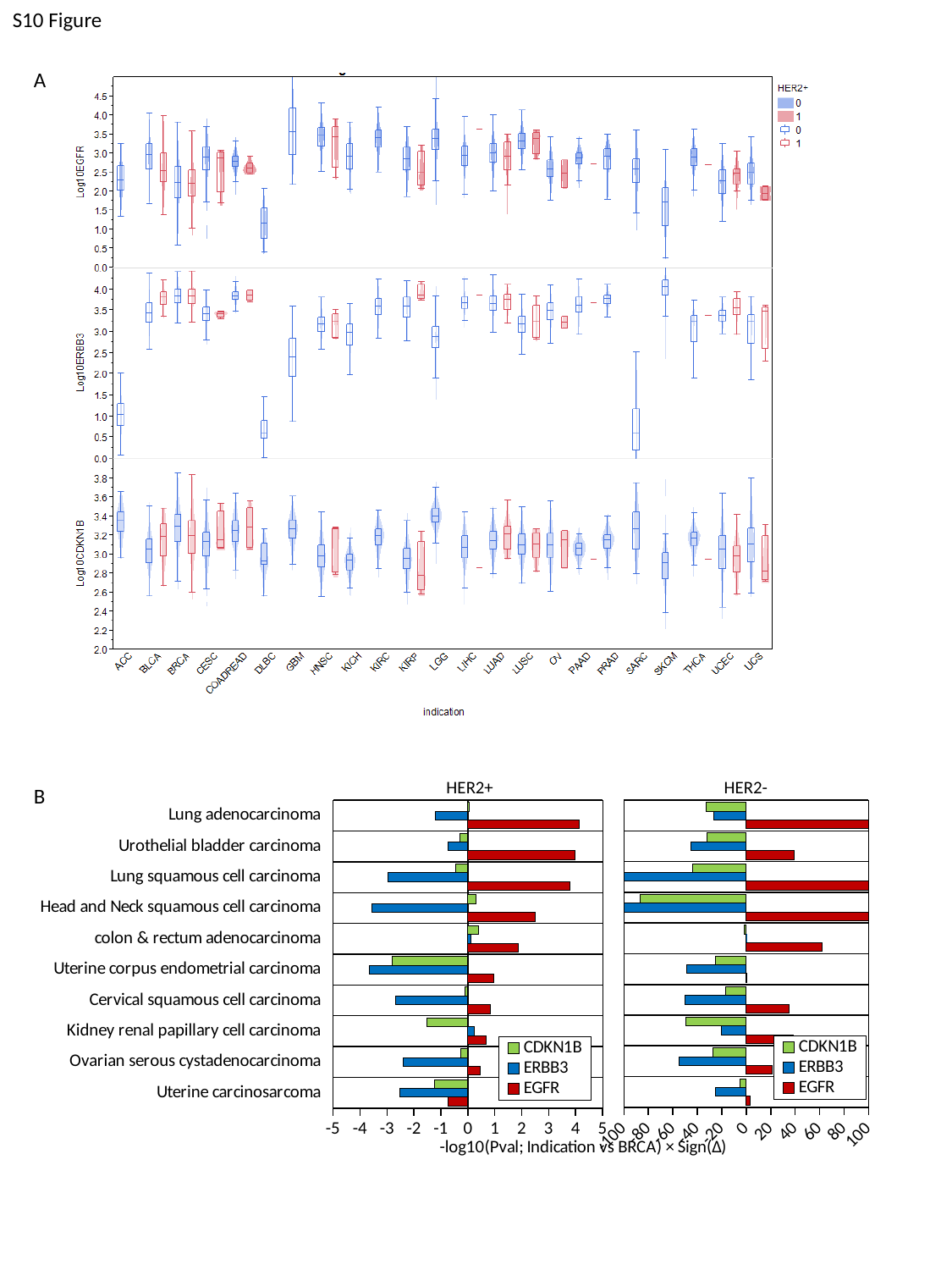

S10 Figure
A
HER2+
HER2-
B
### Chart
| Category | | | |
|---|---|---|---|
| Uterine carcinosarcoma | -0.7488533624811191 | -2.5170991775025087 | -1.2421477480918945 |
| Ovarian serous cystadenocarcinoma | 0.44864752177421297 | -2.383165286112117 | -0.2690070817067257 |
| Kidney renal papillary cell carcinoma | 0.6837676620208007 | 0.2531937533191386 | -1.5193838360078487 |
| Cervical squamous cell carcinoma | 0.8463411049590689 | -2.690394892480273 | -0.11497101705088596 |
| Uterine corpus endometrial carcinoma | 0.9777752985049463 | -3.6533691611769035 | -2.7953579830822317 |
| colon & rectum adenocarcinoma | 1.8754767484845685 | 0.10435902952322484 | 0.39153191384886743 |
| Head and Neck squamous cell carcinoma | 2.510790325440553 | -3.5595078490047145 | 0.2969917930251812 |
| Lung squamous cell carcinoma | 3.7770408745283155 | -2.960581262000489 | -0.45614836217738647 |
| Urothelial bladder carcinoma | 3.971351780949909 | -0.7491095940883288 | -0.3034198350595233 |
| Lung adenocarcinoma | 4.139090775501595 | -1.1990715322004883 | 0.045979458757049785 |
### Chart
| Category | | | |
|---|---|---|---|
| Uterine carcinosarcoma | 3.2060332914613907 | -25.231603457359807 | -5.420954872496748 |
| Ovarian serous cystadenocarcinoma | 20.909900946630827 | -54.988514824339084 | -27.34860635138678 |
| Kidney renal papillary cell carcinoma | 38.74184217414696 | -20.073913437879693 | -49.125811227646295 |
| Cervical squamous cell carcinoma | 34.71756863306313 | -49.906171785706206 | -16.837808742751903 |
| Uterine corpus endometrial carcinoma | 0.24636794064083936 | -48.64906545541817 | -25.044872808288126 |
| colon & rectum adenocarcinoma | 62.107543267988845 | -0.11075188826147439 | -1.7449930879835196 |
| Head and Neck squamous cell carcinoma | 170.39767423156096 | -168.4528025296451 | -86.93675619493091 |
| Lung squamous cell carcinoma | 153.47629289430213 | -164.24385493415593 | -43.77793507931937 |
| Urothelial bladder carcinoma | 39.52229111839235 | -44.99389651227011 | -31.723276959339344 |
| Lung adenocarcinoma | 106.98757905708348 | -26.75695667166012 | -32.59415407894626 |
