# Supplementary material for: HER2+ Cancer Cell Dependence on PI3K vs. MAPK Signaling Axes Is Determined by Expression of EGFR, ERBB3 and CDKN1B
Source: PLoS Comput Biol. 2016 Apr 1;12(4):e1004827. doi: 10.1371/journal.pcbi.1004827 (PMC4818107; doi:10.1371/journal.pcbi.1004827)
Supplement: S1 Table — (DOCX) [file pcbi.1004827.s012.docx]

**Table S1.** Summary of logic-based cell growth models evaluated.

| MODEL | M1 | M2 | M3 | M4 | M5 | M6 | M7 | M8 | M9 |
| --- | --- | --- | --- | --- | --- | --- | --- | --- | --- |
| PROLIFERATION | K | OR | AND | K | K | OR | OR | AND | AND |
| DEATH | K | K | K | OR | AND | OR | AND | OR | AND |
| PARAMETERS | 2 | 6 | 6 | 6 | 6 | 10 | 10 | 10 | 10 |

Wherein:

$$\frac{\boldsymbol{dX}\boldsymbol{1}}{\boldsymbol{dt}}\boldsymbol{=}\boldsymbol{\mu}_{\boldsymbol{MAX}}\boldsymbol{-}\boldsymbol{\delta}_{\boldsymbol{MAX}}$$

$$\frac{\boldsymbol{dX}\boldsymbol{2}}{\boldsymbol{dt}}\boldsymbol{=}\boldsymbol{\mu}_{\boldsymbol{MAX}}\left( \boldsymbol{1-}\frac{\left( \boldsymbol{w}_{\boldsymbol{akt}}\boldsymbol{\cdot AKTi+}\boldsymbol{w}_{\boldsymbol{erk}}\boldsymbol{\cdot MEKi} \right)^{\boldsymbol{k}}}{\boldsymbol{\tau+}\left( \boldsymbol{w}_{\boldsymbol{akt}}\boldsymbol{\cdot AKTi+}\boldsymbol{w}_{\boldsymbol{erk}}\boldsymbol{\cdot MEKi} \right)^{\boldsymbol{k}}} \right)\boldsymbol{-}\boldsymbol{\delta}_{\boldsymbol{MAX}}$$

$$\frac{\boldsymbol{dX}\boldsymbol{3}}{\boldsymbol{dt}}\boldsymbol{=}\boldsymbol{\mu}_{\boldsymbol{MAX}} \left( \mathbf{1}\boldsymbol{-}\frac{\boldsymbol{AKTi}^{\boldsymbol{k\_akt}}}{\boldsymbol{\tau}_{\boldsymbol{akt}}\boldsymbol{+}\boldsymbol{AKT}^{\boldsymbol{k\_akt}}} \right)\boldsymbol{\cdot}\left( \boldsymbol{1-}\frac{\boldsymbol{MEKi}^{\boldsymbol{k\_erk}}}{\boldsymbol{\tau}_{\boldsymbol{erk}}\boldsymbol{+}\boldsymbol{MEK}^{\boldsymbol{k\_erk}}} \right)\boldsymbol{-}\boldsymbol{\delta}_{\boldsymbol{MAX}}$$

$$\frac{\boldsymbol{dX}\boldsymbol{4}}{\boldsymbol{dt}}\boldsymbol{=}\boldsymbol{\mu}_{\boldsymbol{MAX}}\boldsymbol{-}\boldsymbol{\delta}_{\boldsymbol{MAX}}\left( \frac{\left( \boldsymbol{w}_{\boldsymbol{akt}}\boldsymbol{\cdot AKTi+}\boldsymbol{w}_{\boldsymbol{erk}}\boldsymbol{\cdot MEKi} \right)^{\boldsymbol{k}}}{\boldsymbol{\tau+}\left( \boldsymbol{w}_{\boldsymbol{akt}}\boldsymbol{\cdot AKTi+}\boldsymbol{w}_{\boldsymbol{erk}}\boldsymbol{\cdot MEKi} \right)^{\boldsymbol{k}}} \right)$$

$$\frac{\boldsymbol{dX}\boldsymbol{5}}{\boldsymbol{dt}}\boldsymbol{=}\boldsymbol{\mu}_{\boldsymbol{MAX}}\boldsymbol{-}\boldsymbol{\delta}_{\boldsymbol{MAX}}\left( \frac{\boldsymbol{AKTi}^{\boldsymbol{k\_akt}}}{\boldsymbol{\tau}_{\boldsymbol{akt}}\boldsymbol{+}\boldsymbol{AKTi}^{\boldsymbol{k\_akt}}} \right)\boldsymbol{\cdot}\left( \frac{\boldsymbol{MEKi}^{\boldsymbol{k\_erk}}}{\boldsymbol{\tau}_{\boldsymbol{erk}}\boldsymbol{+}\boldsymbol{MEKi}^{\boldsymbol{k\_erk}}} \right)$$

$$\frac{\boldsymbol{dX}\boldsymbol{6}}{\boldsymbol{dt}}\boldsymbol{=}\boldsymbol{\mu}_{\boldsymbol{MAX}}\left( \boldsymbol{1-}\frac{\left( \boldsymbol{w}_{\boldsymbol{akt}}\boldsymbol{\cdot AKTi+}\boldsymbol{w}_{\boldsymbol{erk}}\boldsymbol{\cdot MEKi} \right)^{\boldsymbol{k}}}{\boldsymbol{\tau+}\left( \boldsymbol{w}_{\boldsymbol{akt}}\boldsymbol{\cdot AKTi+}\boldsymbol{w}_{\boldsymbol{erk}}\boldsymbol{\cdot MEKi} \right)^{\boldsymbol{k}}} \right)\boldsymbol{-}\boldsymbol{\delta}_{\boldsymbol{MAX}}\left( \frac{\left( \boldsymbol{w}_{\boldsymbol{akt}}\boldsymbol{\cdot AKTi+}\boldsymbol{w}_{\boldsymbol{erk}}\boldsymbol{\cdot MEKi} \right)^{\boldsymbol{k}}}{\boldsymbol{\tau+}\left( \boldsymbol{w}_{\boldsymbol{akt}}\boldsymbol{\cdot AKTi+}\boldsymbol{w}_{\boldsymbol{erk}}\boldsymbol{\cdot MEKi} \right)^{\boldsymbol{k}}} \right)$$

$$\frac{\boldsymbol{dX}\boldsymbol{7}}{\boldsymbol{dt}}\boldsymbol{=}\boldsymbol{\mu}_{\boldsymbol{MAX}}\left( \boldsymbol{1-}\frac{\left( \boldsymbol{w}_{\boldsymbol{akt}}\boldsymbol{\cdot AKTi+}\boldsymbol{w}_{\boldsymbol{erk}}\boldsymbol{\cdot MEKi} \right)^{\boldsymbol{k}}}{\boldsymbol{\tau+}\left( \boldsymbol{w}_{\boldsymbol{akt}}\boldsymbol{\cdot AKTi+}\boldsymbol{w}_{\boldsymbol{erk}}\boldsymbol{\cdot MEKi} \right)^{\boldsymbol{k}}} \right)\boldsymbol{-}\boldsymbol{\delta}_{\boldsymbol{MAX}}\left( \frac{\left( \boldsymbol{w}_{\boldsymbol{akt}}\boldsymbol{\cdot AKTi+}\boldsymbol{w}_{\boldsymbol{erk}}\boldsymbol{\cdot MEKi} \right)^{\boldsymbol{k}}}{\boldsymbol{\tau+}\left( \boldsymbol{w}_{\boldsymbol{akt}}\boldsymbol{\cdot AKTi+}\boldsymbol{w}_{\boldsymbol{erk}}\boldsymbol{\cdot MEKi} \right)^{\boldsymbol{k}}} \right)$$

$$\frac{\boldsymbol{dX}\boldsymbol{8}}{\boldsymbol{dt}}\boldsymbol{=}\boldsymbol{\mu}_{\boldsymbol{MAX}} \left( \mathbf{1}\boldsymbol{-}\frac{\boldsymbol{AKTi}^{\boldsymbol{k\_akt}}}{\boldsymbol{\tau}_{\boldsymbol{akt}}\boldsymbol{+}\boldsymbol{AKT}^{\boldsymbol{k\_akt}}} \right)\boldsymbol{\cdot}\left( \boldsymbol{1-}\frac{\boldsymbol{MEKi}^{\boldsymbol{k\_erk}}}{\boldsymbol{\tau}_{\boldsymbol{erk}}\boldsymbol{+}\boldsymbol{MEK}^{\boldsymbol{k\_erk}}} \right)\boldsymbol{-}\boldsymbol{\delta}_{\boldsymbol{MAX}}\left( \frac{\left( \boldsymbol{w}_{\boldsymbol{akt}}\boldsymbol{\cdot AKTi+}\boldsymbol{w}_{\boldsymbol{erk}}\boldsymbol{\cdot MEKi} \right)^{\boldsymbol{k}}}{\boldsymbol{\tau+}\left( \boldsymbol{w}_{\boldsymbol{akt}}\boldsymbol{\cdot AKTi+}\boldsymbol{w}_{\boldsymbol{erk}}\boldsymbol{\cdot MEKi} \right)^{\boldsymbol{k}}} \right)$$

$$\frac{\boldsymbol{dX}\boldsymbol{9}}{\boldsymbol{dt}}\boldsymbol{=}\boldsymbol{\mu}_{\boldsymbol{MAX}}\left( \boldsymbol{1-}\frac{\left( \boldsymbol{w}_{\boldsymbol{akt}}\boldsymbol{\cdot AKTi+}\boldsymbol{w}_{\boldsymbol{erk}}\boldsymbol{\cdot MEKi} \right)^{\boldsymbol{k}}}{\boldsymbol{\tau+}\left( \boldsymbol{w}_{\boldsymbol{akt}}\boldsymbol{\cdot AKTi+}\boldsymbol{w}_{\boldsymbol{erk}}\boldsymbol{\cdot MEKi} \right)^{\boldsymbol{k}}} \right)\boldsymbol{-}\boldsymbol{\delta}_{\boldsymbol{MAX}}\left( \frac{\boldsymbol{AKTi}^{\boldsymbol{k\_akt}}}{\boldsymbol{\tau}_{\boldsymbol{akt}}\boldsymbol{+}\boldsymbol{AKTi}^{\boldsymbol{k\_akt}}} \right)\boldsymbol{\cdot}\left( \frac{\boldsymbol{MEKi}^{\boldsymbol{k\_erk}}}{\boldsymbol{\tau}_{\boldsymbol{erk}}\boldsymbol{+}\boldsymbol{MEKi}^{\boldsymbol{k\_erk}}} \right)$$

$$\frac{\boldsymbol{dX}\boldsymbol{10*}}{\boldsymbol{dt}}\boldsymbol{=}\boldsymbol{\mu}_{\boldsymbol{MAX}}\left( \boldsymbol{1-}\frac{\left( \boldsymbol{MEKi} \right)^{\boldsymbol{k\_erk}}}{\boldsymbol{\tau}_{\boldsymbol{erk}}\boldsymbol{+}\left( \boldsymbol{MEKi} \right)^{\boldsymbol{k\_erk}}} \right)\boldsymbol{-}\boldsymbol{\delta}_{\boldsymbol{MAX}}\left( \frac{\boldsymbol{AKTi}^{\boldsymbol{k\_akt}}}{\boldsymbol{\tau}_{\boldsymbol{akt}}\boldsymbol{+}\boldsymbol{AKTi}^{\boldsymbol{k\_akt}}} \right)$$
