# Supplementary material for: HER2+ Cancer Cell Dependence on PI3K vs. MAPK Signaling Axes Is Determined by Expression of EGFR, ERBB3 and CDKN1B
Source: PLoS Comput Biol. 2016 Apr 1;12(4):e1004827. doi: 10.1371/journal.pcbi.1004827 (PMC4818107; doi:10.1371/journal.pcbi.1004827)
Supplement: S2 Table — (DOCX) [file pcbi.1004827.s013.docx]

**Table S2.** “M4” Model Parameters and associated error

| Cell | u_max | d_max | w_akt | w_erk | k | tau | BIAS | MSE |
| --- | --- | --- | --- | --- | --- | --- | --- | --- |
| *Lower Bound* | 1E-05 | 1E-05 | 1E-04 | 1E-04 | 3E-01 | 1E-02 |  |  |
| *Upper Bound* | 5E-02 | 5E-02 | 1E+00 | 1E+00 | 3E+00 | 1E-01 |  |  |
| AU565 | 1.94E-02 | 2.91E-02 | 9.83E-01 | 2.29E-02 | 1.00 | 4.80 | 0.95 | 5.49E-03 |
| AU565+hrg | 1.68E-02 | 6.84E-03 | 1.00E-04 | 6.81E-01 | 0.45 | 1.42 | -1.00 | 1.77E-02 |
| BT474-M3 | 9.19E-03 | 4.75E-02 | 9.91E-01 | 4.77E-03 | 0.76 | 7.47 | 0.99 | 6.38E-03 |
| BT474-M3+hrg | 7.42E-03 | 1.35E-02 | 9.77E-01 | 2.16E-03 | 0.46 | 1.15 | 1.00 | 7.60E-02 |
| CALU3 | 1.72E-02 | 1.23E-02 | 2.38E-03 | 5.35E-01 | 0.92 | 0.01 | -0.99 | 5.69E-02 |
| CALU3+hrg | 2.13E-02 | 1.45E-02 | 1.00E-04 | 9.96E-01 | 1.01 | 0.05 | -1.00 | 2.62E-02 |
| HCC1419 | 7.98E-03 | 5.00E-02 | 9.94E-01 | 6.35E-02 | 0.42 | 63.63 | 0.88 | 1.25E-02 |
| HCC1419+hrg | 1.15E-02 | 9.51E-03 | 1.43E-01 | 9.23E-01 | 2.02 | 0.10 | -0.73 | 1.59E-02 |
| HCC1954 | 1.85E-02 | 1.21E-02 | 4.76E-03 | 9.33E-02 | 3.00 | 0.01 | -0.90 | 3.16E-02 |
| HCC1954+hrg | 2.11E-02 | 1.48E-02 | 4.32E-02 | 7.92E-01 | 2.12 | 0.08 | -0.90 | 2.35E-02 |
| HCC202 | 1.21E-02 | 5.00E-02 | 9.84E-01 | 3.96E-02 | 0.35 | 29.71 | 0.92 | 2.59E-02 |
| HCC202+hrg | 1.45E-02 | 5.00E-02 | 9.96E-01 | 1.17E-01 | 0.38 | 28.43 | 0.79 | 4.38E-02 |
| JIMT1 | 2.85E-02 | 2.30E-02 | 2.22E-04 | 4.75E-01 | 0.58 | 0.01 | -1.00 | 1.10E-01 |
| JIMT1+hrg | 2.81E-02 | 2.07E-02 | 1.01E-01 | 9.71E-01 | 1.26 | 0.26 | -0.81 | 7.18E-02 |
| MDAMB175VII | 1.09E-02 | 1.47E-02 | 8.97E-03 | 1.07E-01 | 3.00 | 0.01 | -0.85 | 2.61E-02 |
| MDAMB175VII+hrg | 1.32E-02 | 1.70E-02 | 1.20E-02 | 1.88E-01 | 1.28 | 0.01 | -0.88 | 1.75E-02 |
| MDAMB361 | 6.03E-03 | 4.63E-02 | 9.58E-01 | 1.46E-02 | 0.79 | 9.79 | 0.97 | 4.88E-03 |
| MDAMB361+hrg | 3.55E-03 | 1.37E-02 | 9.55E-01 | 1.25E-04 | 1.14 | 1.57 | 1.00 | 5.51E-03 |
| MDAMB453 | 1.02E-02 | 4.84E-02 | 9.94E-01 | 7.33E-02 | 0.66 | 18.13 | 0.86 | 3.78E-02 |
| MDAMB453+hrg | 1.54E-02 | 2.17E-02 | 9.95E-01 | 1.53E-01 | 0.55 | 0.60 | 0.73 | 4.66E-02 |
| NCIH2170 | 2.10E-02 | 5.58E-03 | 4.29E-02 | 8.40E-01 | 1.67 | 0.55 | -0.90 | 1.43E-02 |
| NCIH2170+hrg | 2.70E-02 | 1.98E-02 | 1.00E-04 | 8.56E-01 | 0.32 | 10.38 | -1.00 | 2.10E-02 |
| NCIN87 | 1.19E-02 | 1.03E-02 | 1.99E-01 | 9.14E-01 | 1.76 | 0.20 | -0.64 | 1.13E-02 |
| NCIN87+hrg | 1.67E-02 | 1.21E-02 | 4.13E-02 | 6.96E-01 | 1.71 | 0.17 | -0.89 | 1.30E-02 |
| OE19 | 1.70E-02 | 1.18E-02 | 4.55E-02 | 8.21E-01 | 0.94 | 0.20 | -0.89 | 8.36E-03 |
| OE19+hrg | 2.22E-02 | 1.69E-02 | 2.28E-02 | 9.92E-01 | 0.70 | 0.21 | -0.96 | 8.70E-03 |
| OE33 | 2.54E-02 | 2.33E-02 | 8.76E-02 | 7.20E-01 | 1.59 | 0.13 | -0.78 | 3.92E-02 |
| OE33+hrg | 3.26E-02 | 2.73E-02 | 5.38E-02 | 6.86E-01 | 1.31 | 0.11 | -0.85 | 5.57E-02 |
| SKBR3 | 1.95E-02 | 4.12E-02 | 1.00E+00 | 6.02E-02 | 0.35 | 24.68 | 0.89 | 1.74E-02 |
| SKBR3+hrg | 2.10E-02 | 1.11E-02 | 5.79E-02 | 5.70E-01 | 2.21 | 0.08 | -0.82 | 2.39E-02 |
| SKOV3 | 2.02E-02 | 2.25E-02 | 3.46E-01 | 9.19E-01 | 3.00 | 0.22 | -0.45 | 1.40E-01 |
| SKOV3+hrg | 2.13E-02 | 2.28E-02 | 9.93E-03 | 4.00E-02 | 3.00 | 0.01 | -0.60 | 1.72E-01 |
| ZR751 | 1.65E-02 | 2.67E-02 | 1.00E+00 | 2.50E-02 | 0.30 | 0.14 | 0.95 | 5.69E-02 |
| ZR751+hrg | 2.25E-02 | 1.23E-02 | 9.69E-04 | 7.53E-01 | 1.06 | 0.31 | -1.00 | 1.6E-1 |
| ZR7530 | 6.71E-03 | 2.44E-02 | 1.00E+00 | 1.03E-04 | 0.46 | 12.86 | 1.00 | 4.85E-03 |
| ZR7530+hrg | 6.49E-03 | 5.00E-02 | 7.20E-01 | 6.08E-03 | 0.64 | 24.47 | 0.98 | 2.4E-1 |
