# Supplementary material for: HER2+ Cancer Cell Dependence on PI3K vs. MAPK Signaling Axes Is Determined by Expression of EGFR, ERBB3 and CDKN1B
Source: PLoS Comput Biol. 2016 Apr 1;12(4):e1004827. doi: 10.1371/journal.pcbi.1004827 (PMC4818107; doi:10.1371/journal.pcbi.1004827)
Supplement: S3 Table — A) Capture antibodies used in Luminex assays. B) Detection antibodies used in Luminex assays. (DOCX) [file pcbi.1004827.s014.docx]

**Table S3A.** Capture antibodies used in Luminex assays

| Target | Total/phosphor | Source | Cat. No. |
| --- | --- | --- | --- |
| AKT | Total | R&D Biosystems | DYC887B |
| AKT | pT308 | Cell Signaling Tech. | 4056BF |
| AKT1 | pS473 | Millipore | 05-669 |
| CDKN1B | Total | R&D Biosystems | DYC2256E |
| EGFR | Total | Thermo Fisher | MS-609 |
| EGFR | Total | Thermo Fisher | MS-396 |
| ERBB2a | Total | BioLegend | 324402 |
| ERBB2b | Total | R&D Biosystems | MAB1129 |
| ERBB2c | Total | R&D Biosystems | DYC1768E |
| ERBB3a | Total | R&D Biosystems | MAB3481 |
| ERBB3b | Total | R&D Biosystems | DYC1769E |
| ERBB4a | Total | Thermo Fisher | MS-270 |
| ERBB4b | Total | R&D Biosystems | DYC2115E |
| ERK1/2 | pT202Y204 | Cell Signaling Tech. | 4370BF |
| ERK1/2 | Total | Cell Signaling Tech. | 3374BF |

**Table S3B.** Detection antibodies used in Luminex assays

| Target | Total/phosphor | Source | Cat. No. |
| --- | --- | --- | --- |
| ERK1/2 | Total | Cell Signaling Tech. | 4695BF |
| EGFR | Total | Cell Signaling Tech. | 6627 |
| ERBB2 | Total | R&D Biosystems | BAF1129 |
| ERBB3 | Total | R&D Biosystems | BAM348 |
| ERBB4 | Total | R&D Biosystems | BAF1131 |
| AKT | Total | R&D Biosystems | DYC1775 |
| CDKN1B | Total | R&D Biosystems | DYC2256 |
| Tyrosine | Phospho | Millipore | 16-103 |
