# Supplementary material for: HER2+ Cancer Cell Dependence on PI3K vs. MAPK Signaling Axes Is Determined by Expression of EGFR, ERBB3 and CDKN1B
Source: PLoS Comput Biol. 2016 Apr 1;12(4):e1004827. doi: 10.1371/journal.pcbi.1004827 (PMC4818107; doi:10.1371/journal.pcbi.1004827)
Supplement: S4 Table — (DOCX) [file pcbi.1004827.s015.docx]

**Table S4.** Logistic model parameters, wherein median values and standard deviations are taken from the 36 LOOCV estimates

| β_0_ (Intercept) | β_1_ (CDKNIB) | β_2_ (EGFR) | β_3_ (ERBB3b) |
| --- | --- | --- | --- |
| 1.678 ± 0.191 | -01.43×10^-3^ ± 3.78×10^-4^ | 2.53×10^-4^ ± 3.72×10^-5^ | -3.42×10^-3^ ± 4.39×10^-4^ |
