# Supplementary material for: HER2+ Cancer Cell Dependence on PI3K vs. MAPK Signaling Axes Is Determined by Expression of EGFR, ERBB3 and CDKN1B
Source: PLoS Comput Biol. 2016 Apr 1;12(4):e1004827. doi: 10.1371/journal.pcbi.1004827 (PMC4818107; doi:10.1371/journal.pcbi.1004827)
Supplement: S5 Table — (DOCX) [file pcbi.1004827.s016.docx]

**Table S5.**  Raw Protein Signals, and Logistic Model Predictions of *Pathway Bias*

| Cell | CDKN1B | EGFRa | ERBB3b | LOOCV_model | FIT_model | DATA |
| --- | --- | --- | --- | --- | --- | --- |
| AU565 | 188.5 | 10306.75 | 1562.25 | 0.52 | 0.73 | 0.98 |
| BT474-M3 | 357 | 2937.75 | 548.5 | 0.15 | 0.23 | 1.00 |
| CALU3 | -277.5 | 15817.25 | 246.5 | -0.98 | -0.98 | -1.00 |
| HCC1419 | 1141 | 53.25 | 671.25 | 0.87 | 0.88 | 0.94 |
| HCC1954 | 5.75 | 13705.25 | 779.5 | -0.72 | -0.75 | -0.95 |
| HCC202 | 776.25 | 4328.5 | 388.25 | -0.02 | 0.09 | 0.96 |
| JIMT1 | -204 | 16023 | 177.5 | -0.99 | -0.99 | -1.00 |
| MDAMB175VII | 56 | 606.5 | 133.25 | -0.22 | -0.37 | -0.92 |
| MDAMB361 | 620.75 | 631.5 | 198.5 | 0.03 | 0.12 | 0.98 |
| MDAMB453 | 720 | 214.5 | 753.5 | 0.82 | 0.83 | 0.93 |
| NCIH2170 | 352.25 | 996.75 | 116.5 | -0.15 | -0.25 | -0.95 |
| NCIN87 | 90.25 | 15080.25 | 139 | -0.98 | -0.98 | -0.78 |
| OE19 | 110.5 | 8909.75 | 489.5 | -0.64 | -0.66 | -0.94 |
| OE33 | -90 | 8836.75 | 27.75 | -0.94 | -0.94 | -0.88 |
| SKBR3 | 219.25 | 9863.75 | 335.75 | -0.95 | -0.80 | 0.94 |
| SKOV3 | -21.75 | 7840 | 22 | -0.91 | -0.91 | -0.62 |
| ZR751 | 1378.75 | 1308.5 | 268 | 0.46 | 0.60 | 0.98 |
| ZR7530 | 866 | 31.5 | 1240 | 0.97 | 0.97 | 1.00 |
| AU565+hrg | 254.25 | 11444 | 1007.5 | 0.22 | -0.13 | -1.00 |
| BT474-M3+hrg | 458.25 | 2845.25 | 589 | 0.32 | 0.37 | 1.00 |
| CALU3+hrg | -2.5 | 16327.25 | 292 | -0.97 | -0.97 | -1.00 |
| HCC1419+hrg | 1137 | 48.5 | 619.75 | 0.97 | 0.86 | -0.85 |
| HCC1954+hrg | 107.25 | 14314.75 | 837 | -0.65 | -0.71 | -0.95 |
| HCC202+hrg | 926.25 | 4586.5 | 387.25 | 0.00 | 0.16 | 0.88 |
| JIMT1+hrg | -191.75 | 15704.25 | 139.5 | -0.99 | -0.99 | -0.90 |
| MDAMB175VII+hrg | 52 | 524.75 | 135.5 | -0.20 | -0.36 | -0.94 |
| MDAMB361+hrg | 295.75 | 533.5 | 187.5 | -0.30 | -0.12 | 1.00 |
| MDAMB453+hrg | 769.25 | 240.5 | 387 | 0.50 | 0.54 | 0.85 |
| NCIH2170+hrg | 118.25 | 1105 | 68.25 | -0.39 | -0.48 | -1.00 |
| NCIN87+hrg | 167.5 | 15084.75 | 165.75 | -0.97 | -0.97 | -0.94 |
| OE19+hrg | 192 | 9607.5 | 593.5 | -0.53 | -0.57 | -0.98 |
| OE33+hrg | -115.5 | 9719.25 | 36.75 | -0.95 | -0.95 | -0.92 |
| SKBR3+hrg | 206 | 9712.75 | 177.5 | -0.87 | -0.88 | -0.90 |
| SKOV3+hrg | -14 | 8960.5 | 26.75 | -0.93 | -0.93 | -0.75 |
| ZR751+hrg | 1240.25 | 1609.75 | 166.5 | 0.85 | 0.37 | -1.00 |
| ZR7530+hrg | 841.25 | 34 | 921 | 0.92 | 0.92 | 0.99 |
