# Supplementary material for: HER2+ Cancer Cell Dependence on PI3K vs. MAPK Signaling Axes Is Determined by Expression of EGFR, ERBB3 and CDKN1B
Source: PLoS Comput Biol. 2016 Apr 1;12(4):e1004827. doi: 10.1371/journal.pcbi.1004827 (PMC4818107; doi:10.1371/journal.pcbi.1004827)
Supplement: S6 Table — (DOCX) [file pcbi.1004827.s017.docx]

**Table S6:** Evaluation of EGFR, ERBB3, pAKT, and pERK protein expression as predictive biomarkers for HDACi, PI3K/AKT/MTORi, and MEKi sensitivity in HER2+ cell represented in the GDSC database.

| TARGETS | EGFR | ERBB3 | pAKT | pERK | EGFR & ERBB3 | EGFR^HI^ v EGFR^LO^ERBB3^HI^* |
| --- | --- | --- | --- | --- | --- | --- |
| HDACi (2) | 1 | 0 | 0 | 0 | 1 | 1 |
| PI3K/AKT/MTORi (11) | 1 | 0 | 1 | 0 | 0 | 2 |
| MEKi (4) | 2 | 0 | 0 | 0 | 0 | 2 |
| HITS @ *P* < 0.1 (138) | 9 | 9 | 9 | 8 | 6 | 13 |
| P-VAL** (HDACi) | 0.13 | 1 | 1 | 1 | 0.085 | 0.18 |
| P-VAL (P/A/Mi) | 0.54 | 1 | 0.54 | 1 | 1 | 0.28 |
| P-VAL (MEKi) | 0.021 | 1 | 1 | 1 | 1 | 0.044 |

PI3Ki: AZD6482, GDC0941, NVPBEZ235, WO2009093972

AKTi: A443654, AKTinhibitorVIII, MK2206

MOTRi: AZD8055, JW7521, Temsirolimus, Rapamycin

MEKi: PD-0325901, CI-1040, AZD6244, RDEA119

*ERBB3^HI^ defined as 30^th^ percentile

**Hypergeometric P-values
